# Dietary-derived vitamin B12 protects *Caenorhabditis elegans* from thiol-reducing agents

**DOI:** 10.1186/s12915-022-01415-y

**Published:** 2022-10-08

**Authors:** Alan D. Winter, Elissa Tjahjono, Leonardo J. Beltrán, Iain L. Johnstone, Neil J. Bulleid, Antony P. Page

**Affiliations:** 1 School of Biodiversity, One Health and Veterinary Medicine, University of Glasgow, Glasgow, G61 1QH UK; 2 School of Molecular Biosciences, University of Glasgow, Glasgow, G12 8QQ UK

**Keywords:** Vitamin B12, Reductive stress, DTT, Methyltransferase, Methionine

## Abstract

**Background:**

One-carbon metabolism, which includes the folate and methionine cycles, involves the transfer of methyl groups which are then utilised as a part of multiple physiological processes including redox defence. During the methionine cycle, the vitamin B12-dependent enzyme methionine synthetase converts homocysteine to methionine. The enzyme S-adenosylmethionine (SAM) synthetase then uses methionine in the production of the reactive methyl carrier SAM. SAM-binding methyltransferases then utilise SAM as a cofactor to methylate proteins, small molecules, lipids, and nucleic acids.

**Results:**

We describe a novel SAM methyltransferase, RIPS-1, which was the single gene identified from forward genetic screens in *Caenorhabditis elegans* looking for resistance to lethal concentrations of the thiol-reducing agent dithiothreitol (DTT). As well as RIPS-1 mutation, we show that in wild-type worms, DTT toxicity can be overcome by modulating vitamin B12 levels, either by using growth media and/or bacterial food that provide higher levels of vitamin B12 or by vitamin B12 supplementation. We show that active methionine synthetase is required for vitamin B12-mediated DTT resistance in wild types but is not required for resistance resulting from RIPS-1 mutation and that susceptibility to DTT is partially suppressed by methionine supplementation. A targeted RNAi modifier screen identified the mitochondrial enzyme methylmalonyl-CoA epimerase as a strong genetic enhancer of DTT resistance in a RIPS-1 mutant. We show that RIPS-1 is expressed in the intestinal and hypodermal tissues of the nematode and that treating with DTT, β-mercaptoethanol, or hydrogen sulfide induces RIPS-1 expression. We demonstrate that RIPS-1 expression is controlled by the hypoxia-inducible factor pathway and that homologues of RIPS-1 are found in a small subset of eukaryotes and bacteria, many of which can adapt to fluctuations in environmental oxygen levels.

**Conclusions:**

This work highlights the central importance of dietary vitamin B12 in normal metabolic processes in *C. elegans*, defines a new role for this vitamin in countering reductive stress, and identifies RIPS-1 as a novel methyltransferase in the methionine cycle.

**Supplementary Information:**

The online version contains supplementary material available at 10.1186/s12915-022-01415-y.

## Background

**Fig. 1:**
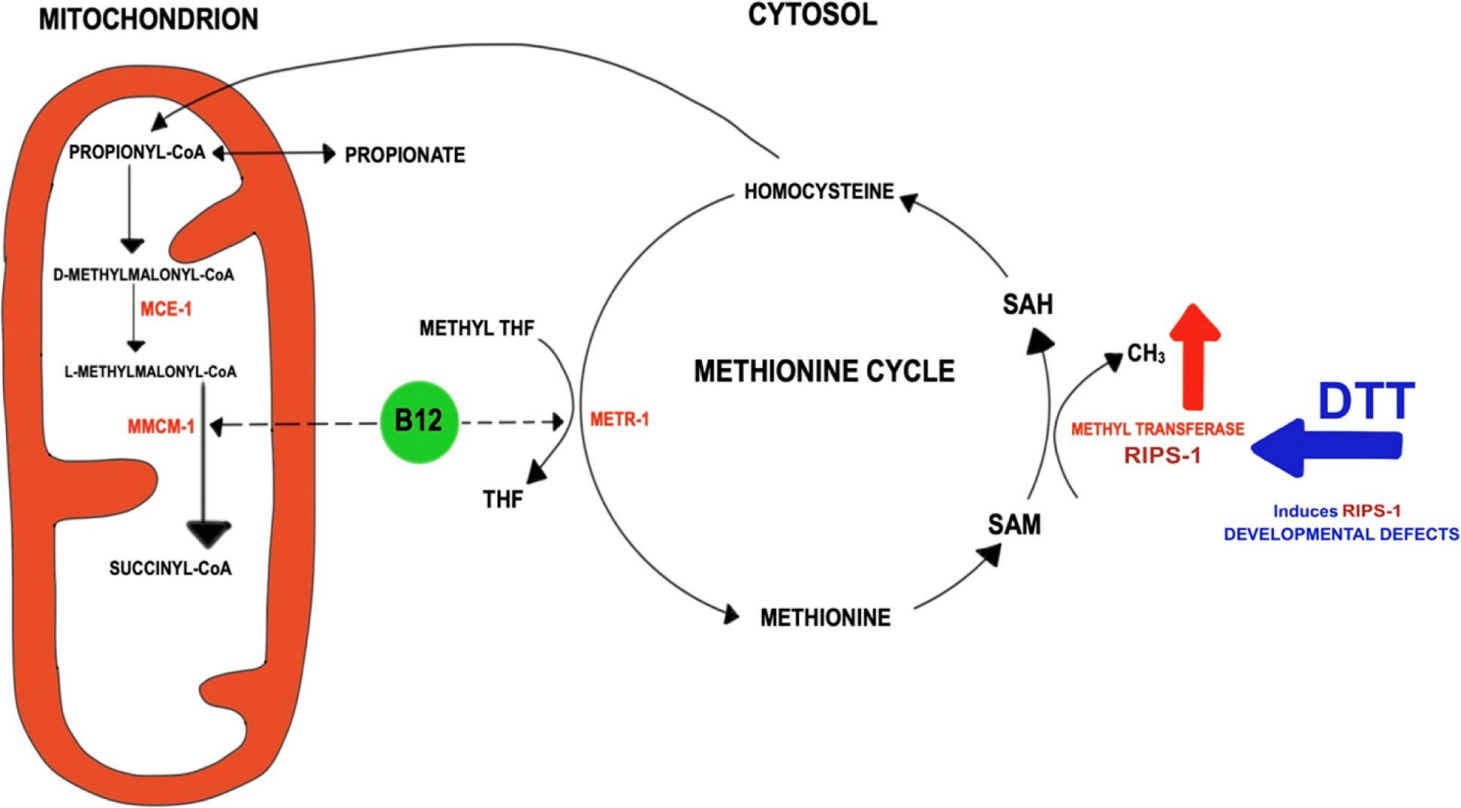
Model for the role of RIPS-1 methyltransferase in the mitochondrial and cytosolic vitamin B12-dependent pathways. Adenosyl cobalamin (B12) is required in the conversion of TCA cycle-dependent propionyl-CoA to succinyl-CoA. This process includes the isomerisation of d-methylmalonyl-CoA to l-methylmalonyl-CoA and is catalysed by the enzyme methylmalonyl-CoA epimerase (MCE-1), followed by the B12-dependent methyl malonyl-CoA mutase (MMCM-1) conversion to succinyl-CoA that feeds directly into the TCA cycle. In conditions where B12 is depleted or MCE-1 or MMCM-1 are disrupted, this pathway is interrupted and propionyl-CoA is converted into toxic propionate (and the *acdh-1*^*prom*^::GFP marker is induced). Methyl cobalamin (vitamin B12) is also required for the folate and methionine cycles in the cytosol by acting as an essential co-factor for the methyltetrahydrofolate-homocysteine methyltransferase (methionine synthetase) enzyme (METR-1). This enzyme demethylates methyl tetrahydrofolate (methyl THF) to tetrahydrofolate (THF) in the folate cycle and methylates homocysteine to methionine in the methionine cycle. Methionine can be further converted to *S*-adenosyl methionine (SAM) then gets demethylated to *S*-adenosyl homocysteine (SAH). This demethylation reaction is a major source of methyl groups that is catalysed by *S*-adenosyl methionine methyltransferases including RIPS-1. The final step of the methionine cycle involves the conversion of SAH to homocysteine, which can ultimately feed into the TCA cycle via cystathionine, alpha-ketobutyrate, and propionyl-CoA. Thiol-reducing agents, including DTT, induce the *S*-adenosyl methionine methyltransferase RIPS-1. The vitamin B12-dependent reactions including methionine synthetase become rate-limiting, and in the absence of B12 supplementation, thiol-reducing agents cause developmental defects and lethality in *C. elegans*

Cobalamin, or vitamin B12, is made by a limited subset of prokaryotes but is required by a wide range of eukaryotes including the nematode *Caenorhabditis elegans* and mammals [1]. This essential vitamin exists in two biologically active forms, methyl cobalamin, an indispensable cytosolic cofactor for methionine synthetase, and the mitochondrially required adenosyl cobalamin, a critical cofactor for methylmalonyl coenzyme A mutase (MMCM) [2]. In *C. elegans*, the cytosolic vitamin B12-dependent methionine synthetase METR-1 converts homocysteine to methionine, the main provider of methyl groups for numerous methylation processes. Meanwhile, the mitochondrial enzyme MMCM-1 converts methylmalonyl-CoA to succinyl-CoA, a reaction which follows an epimerase reaction catalysed by methylmalonyl-CoA epimerase (MCE-1) (Fig. 1). These two functions are required in both nematodes and humans; chronic B12 deficiency in worms results in growth defects, infertility, and reduced lifespan [3], while in humans, vitamin B12 deficiency leads to megaloblastic anaemia and neurological problems [2].

Bacterial diet can have a profound effect on the development of *C. elegans* and vitamin B12 has been shown to be an essential vitamin [4]. *C. elegans* acquires vitamin B12 from its bacterial food source; however, the most commonly used laboratory food source, the *E. coli* strain OP50, is mildly deficient in vitamin B12 when compared to HT115 (used for *C. elegans* RNAi feeding experiments) and other bacterial species such as *Comamonas* [5–8]. Mild vitamin B12 deficiency affects the conversion of methylmalonyl coenzyme A to succinyl-CoA in the mitochondrion and impairs the breakdown of propionate and branched-chain amino acids, ultimately leading to mitochondrial dysfunction. A toxic build-up of propionate induces acyl-CoA dehydrogenase (ACDH) thereby activating a propionate shunt pathway, as shown in *C. elegans* using the transgenic *acdh-1*^*prom*^::GFP reporter which is a highly sensitive indicator of vitamin B12 deficiency [4].

Thiol-reducing agents such as dithiothreitol (DTT) and β-mercaptoethanol represent powerful reducing agents that break disulfide bonds in proteins. While forward genetic screens have been performed to determine modes of resistance to oxidation [9], there is limited information regarding the mechanisms of resistance to reducing agents. In this study, we have applied the genetically amenable model *C. elegans* to help elucidate the mechanisms of resistance to reductive stress in a metazoan organism. This study reveals that resistance to thiol-reducing agents is a vitamin B12-dependent process involving the methionine cycle in the cytosol and the TCA cycle in the mitochondria. Genetic screens for DTT resistance identified a single SAM methyltransferase, R08E5.3, loss of which affords protection from reductive stress. While this manuscript was in preparation, a different group independently reported the role of R08E5.3 in DTT resistance and isolated a group of 10 different mutations in the same methyltransferase gene [10]. This group also connected this resistance to the methionine cycle and highlighted the importance of vitamin B12 in the resistance phenotype [10]. WormBase release 285 lists R08E5.3 as RIPS-1 (for *Rhy-1*-interacting protein in sulfide response), and we use this gene name throughout. We show that RIPS-1 expression is induced by thiol-reducing agents and hydrogen sulfide (H_2_S) and by disruption of negative regulators of hypoxia-inducible factor 1 (HIF-1), including *rhy-1*. RIPS-1 induction is dependent on the transcription factor HIF-1 which represents the main oxygen sensing system in multicellular animals. Phylogenetic analysis reveals that RIPS-1 belongs to a group of methyltransferases that in metazoans is found only in species that can switch between aerobic and anaerobic metabolisms.

## Results

### Survival on the reducing agent DTT is dependent on bacterial food and growth media

**Fig. 2:**
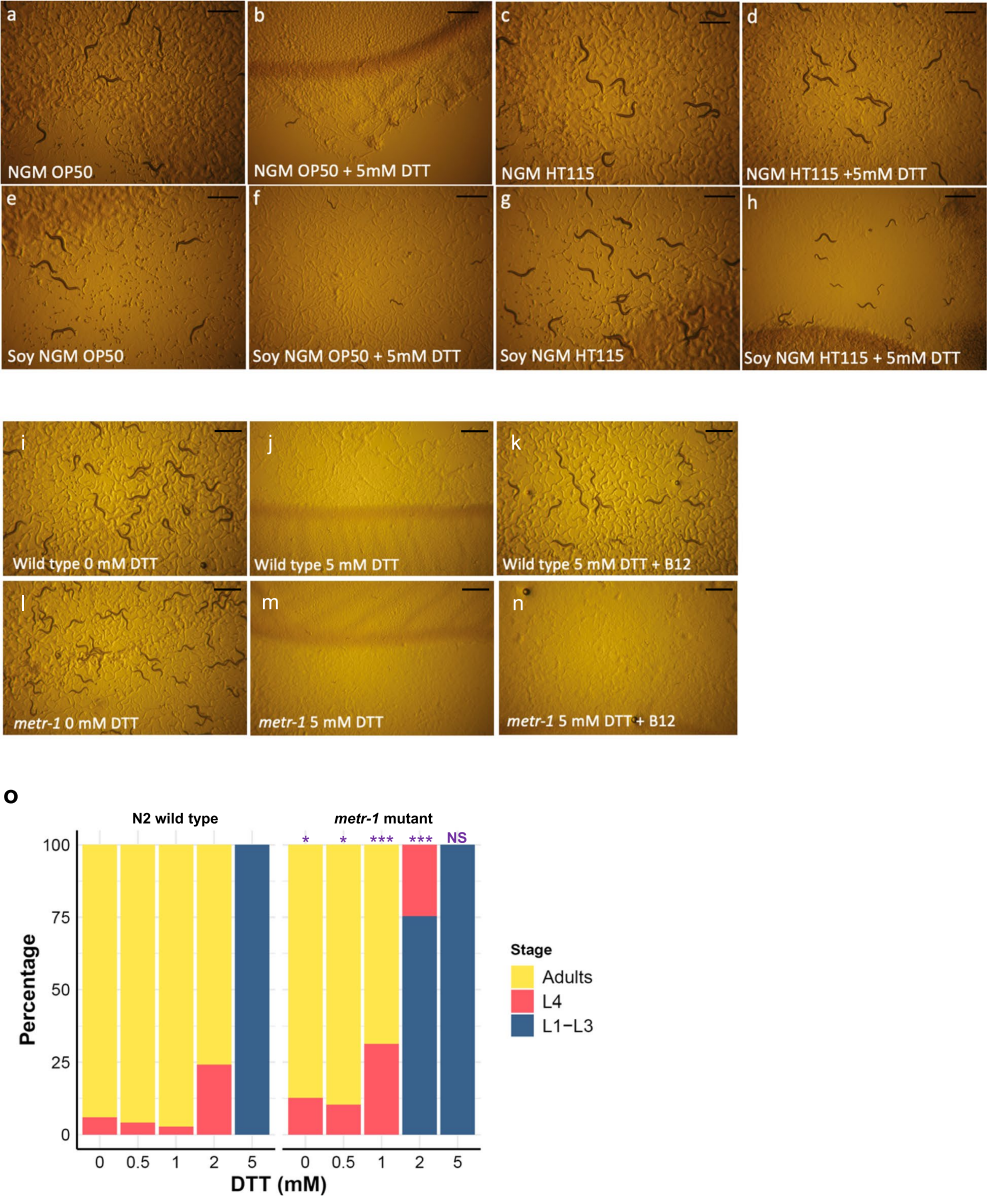
Survival on DTT is influenced by vitamin B12 availability and is METR-1-dependent. **a**–**h** Plate images of wild-type *C. elegans* (N2) on animal-derived peptone NGM agar (**a**–**d**) or on soy-derived NGM agar (**e**–**h**). Worms were reared on OP50-1 (**a**, **e**), with OP50-1 plus 5 mM DTT (**b**, **f**), with HT115(DE3) (**c**, **g**), or with HT115(DE3) plus 5 mM DTT (**d**, **h**). Wild-type (N2) embryos were added at day 0, and images were taken at day 4. **i**–**n** Development of wild-type *C. elegans* (N2) embryos (**i**–**k**) or *C. elegans metr-1*(*ok521*) mutant embryos (**l**–**n**) over 4 days on soybean peptone NGM agar and *E. coli* strain OP50-1 in the absence of DTT (**i**, **l**), in the presence of 5 mM DTT (**j**, **m**), or in the presence of 5 mM DTT plus 64 nM vitamin B12 (**k**, **n**). Scale bars for all images denote 1 mm. **o** Plotted percentage development of wild-type (N2) or *metr-1* mutant worms to adulthood after 4 days of treatment with no DTT (*n* = N2: 202, *metr-1*: 142), 0.5 mM DTT (*n* = N2: 240, *metr-1*: 174), 1 mM DTT (*n* = N2: 292, *metr-1*: 134), 2 mM DTT (*n* = N2: 290, *metr-1*: 162), or 5 mM DTT (*n* = N2: 228, *metr-1*: 116). *p*-values were determined from Fisher’s exact test. NS, not significant, *p < 0.05, ***p < 0.001. Purple significance marks indicate comparison of *metr-1* mutant to N2 wild type for each treatment group

Due to our long-standing interest in the control of cellular redox, we examined the effect of thiol-reducing agents such as dithiothreitol (DTT) on *C. elegans* development. During these experiments, we made the unexpected observation that both bacterial diet and growth media had a dramatic influence on worm survival in the presence of DTT (Fig. 2a–h). On standard nematode growth media (NGM), wild-type worms that were fed *E. coli* OP50-1, the most frequently used food source, stopped development as larvae in the presence of 5 mM DTT (Fig. 2b). In contrast, wild-type worms that were fed with *E. coli* HT115(DE3) developed to fertile adults in the presence of the same concentration of DTT (Fig. 2d), being almost indistinguishable from worms grown in the absence of DTT on either bacterial food source (Fig. 2a, c).

Major differences identified between the *E. coli* strains OP50-1 and HT115(DE3) are the levels of carbohydrate, fatty acids [11], and vitamin B12. The *E. coli* strain OP50-1 poorly expresses TonB, a transporter required for vitamin B12 uptake from the environment [12], causing this strain to be relatively deficient in vitamin B12 compared to HT115(DE3) [5–8]. To examine this possible link between vitamin B12 and DTT resistance, we tested the effect of different growth media. Standard NGM uses peptone from animal sources that contain vitamin B12, while NGM made from plant-based soybean peptone is deficient in B12. The effect of media on DTT resistance was most noticeable when worms were reared on HT115(DE3) (higher vitamin B12) where in the presence of DTT worm survival on soybean peptone NGM was reduced compared to standard NGM (Fig. 2d, h). As the DTT assay appeared to be influenced by vitamin B12, we used soy peptone NGM and *E. coli* OP50-1 in all subsequent experiments (except for RNAi) as this provided low levels of vitamin B12 from both the growth media and the bacterial food source.

### Supplementation with vitamin B12 results in METR-1-dependent survival on DTT

To define the role of vitamin B12 on the survival of wild-type worms under reductive stress, we compared worm growth using soy peptone NGM and *E. coli* OP50-1 plates supplemented with cyanocob(III)alamin (vitamin B12) to those with no supplementation. Cyanocob(III)alamin is converted to both methylcobalamin, the cytoplasmic form of vitamin B12, and adenosylcobalamin, the mitochondrial form of vitamin B12. Supplementation with 64 nM vitamin B12 completely alleviated the DTT sensitivity of wild-type worms (Fig. 2i–k).

Vitamin B12 is used in only two enzymatic reactions; the conversion of homocysteine and methyl-tetrahydrofolate to methionine and tetrahydrofolate by the cytoplasmic methionine synthetase (METR-1 in *C. elegans*) and in the conversion of methylmalonyl-CoA to succinyl-CoA catalysed by the mitochondrial methylmalonyl-CoA mutase (MMCM-1). To determine if the effect of vitamin B12 required either of these two enzymes, we examined the DTT survival of *metr-1* (Fig. 2l–n) and *mmcm-1* (Additional file 1: Fig. S1) mutants. *metr-1* mutants were susceptible to DTT toxicity even when supplemented with 64 nM vitamin B12 (Fig. 2n, Additional file 1: Fig. S1D-F, M), while *mmcm-1* mutants were resistant to DTT toxicity on B12-supplemented DTT plates (Additional file 1: Fig. S1G-I, M). We conclude that survival on DTT is dependent on vitamin B12 and active METR-1.

We next examined if a *metr-1* mutant displayed enhanced susceptibility to the effects of DTT. Titration of DTT from 0.5 to 5 mM demonstrated that the *metr-1* mutant strain is more sensitive to DTT than the wild-type N2 strain (Fig. 2o). At 2 mM DTT, 76% of wild-type N2 but no *metr-1* mutants developed to adults. *metr-1* mutants are therefore hypersensitive to DTT toxicity.

### Loss-of-function mutations in the methyltransferase RIPS-1 confers resistance to DTT

A major advantage of the *C. elegans* model system is the ability to perform unbiased mutagenic screens. We reasoned that as DTT susceptibility is dependent on both vitamin B12 and B12-utilising enzyme METR-1, a screen aiming to identify DTT resistance mutants may uncover genes influencing either B12 availability, METR-1 function, or the methionine cycle. We therefore used EMS mutagenesis to isolate 14 independent strains that were resistant to 5 mM DTT and analysed nine of these using a combined SNP-based mapping and whole-genome re-sequencing protocol [13]. A strong genetic mapping signal to a 2–3-Mb region on the left-hand side of chromosome V was found for eight strains (mapping for two strains is shown in Additional file 1: Fig. S2A and B). Within this region, the NGS data revealed that all eight strains contained homozygous variants for the methyltransferase *rips-1* (Additional file 1: Fig. S2C and Additional file 2: Table S1). We confirmed these eight alleles using conventional Sanger sequencing and then used Sanger sequencing only for the remaining five DTT-resistant alleles, all of which contained homozygous variants in *rips-1*. Therefore, from our mutagenesis screen, we defined 13 *rips-1* alleles representing 11 unique variants, comprising six amino acid substitutions, four premature stop mutations, and a splice-acceptor site mutation (Additional file 1: Fig. S2C and Additional file 2: Table S1). We also analysed the *rips-1* allele *gk90219314* from the *C. elegans* Million Mutations Project [14] and confirmed that it was also resistant to 5 mM DTT.

We then used RNAi with a 997-bp *rips-1* fragment from the Ahringer lab *C. elegans* RNAi library [15] to confirm that *rips-1* was the gene conferring DTT resistance. We included three *rips-1* paralogues, identified by InParanoid 8 [16] and from our own BLAST analysis (Additional file 3). Only wild-type worms treated with either *rips-1* RNAi or *R08E5.1* RNAi (Additional file 1: Fig. S3A) survived in the presence of 5 mM DTT (Additional file 1: Fig. S3B). It is likely that the *R08E5.1* RNAi survival is the result of an off-target RNAi effect on *rips-1* as both genes share regions of high nucleotide conservation (Additional file 1: Fig. S3A). Finally, we introduced a wild-type *rips-1* transgene into a *rips-1* mutant and found this restored DTT sensitivity (results described in a later section).

**Fig. 3:**
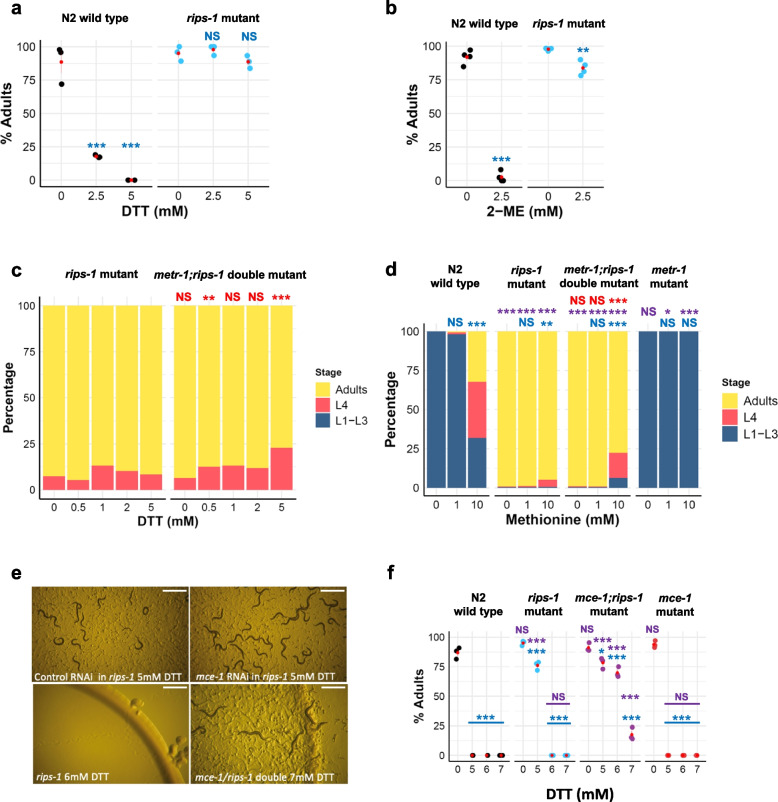
*rips-1* mutants are resistant to thiol-reducing agents, independent of METR-1 and enhanced by loss of MCE-1 (**a**, **b**). Plotted percentage of adults of wild-type (N2) and DTT-resistant *rips-1*(*ij109*) mutant. Embryos were placed on soy peptone NGM agar plates with OP50-1 as food source, supplemented with 0 mM DTT (*n* = N2: 292, *rips-1*: 265), 2.5 mM DTT (*n* = N2: 305, *rips-1*: 268), or 5 mM DTT (*n* = N2: 292, *rips-1*: 261) (**a**), or with 0 mM β-mercaptoethanol (2-ME) (*n* = N2: 164, *rips*: 220) or 2.5 mM 2-ME (*n* = N2: 200, *rips-1*: 227) (**b**). **c ***rips-1*(*ij109*) mutant embryos were assessed for development to adults on 0 mM DTT (*n* = 205), 0.5 mM DTT (*n* = 190), 1 mM DTT (*n* = 244), 2 mM DTT (*n* = 176), or 5 mM DTT (*n* = 214) and compared to *metr-1;rips-1* double mutant [TP391, *metr-1*(*ok521*);*rips-1*(*ij109*)] on 0 mM DTT (*n* = 280), 0.5 mM DTT (*n* = 272), 1 mM DTT (*n* = 260), 2 mM DTT (*n* = 270), or 5 mM DTT (*n* = 210). **d** Development of wild-type (N2), *rips-1*(*ij109*), *metr-1*(*ok521*);*rips-1*(*ij109*), and *metr-1*(*ok521*) worms on 5 mM DTT OP50-1 plates supplemented with 0, 1, or 10 mM methionine. The number of N2, *rips-1* mutant, *metr-1;rips-1* mutant, and *metr-1* mutant animals used in this experiment in this order are as follows: no methionine (*n* = 166, 244, 303, 217), 1 mM methionine (*n* = 259, 279, 259, 292), 10 mM methionine (*n* = 213, 176, 125, 140). **e** RNAi by feeding was performed in a *rips-1*(*ij109*) mutant with control (L4440) or *mce-1* RNAi followed by selection on 5 mM DTT plates. Enhanced resistance was confirmed for a double mutant on DTT, as *rips-1*(*ij109*) single mutant could not survive on 6 mM DTT (bottom left) while *mce-1*(*ok243*);*rips-1*(*ij109)* double mutant survived up to 7 mM DTT (bottom right). Scale bars denote 1 mm. **f** Percentages of adults of N2 wild type and various mutants on 0, 5, 6, and 7 mM DTT. The numbers of animals used in this experiment in each treatment group (0, 5, 6, and 7 mM, respectively) are as follows: N2—*n* = 245, 206, 129, and 206; *rips-1*—*n* = 202, 243, 173, and 267; *mce-1;rips-1*—*n* = 305, 253, 305, and 259; *mce-1*—*n* = 225, 206, 195, and 214; *metr-1*—*n* = 252, 308, 257, and 261; and *mmcm-1*—*n* = 184, 191, 148, and 123. Percentage survival to adulthood was determined after 4 days of treatment. In **a**, **b**, and **f**, red points with lines denote the mean and SEM. For **a** and **f**, *p*-values were determined from two-way ANOVA, followed by Dunnett’s test. For **b**, *p*-values were determined with the two-tailed *t*-test. For **c** and **d**, *p*-values were determined from Fisher’s exact test. NS, not significant, *p < 0.05, **p < 0.01, ***p < 0.001. For all panels, purple significance marks indicate the comparison of mutant worm strains to N2 wild type for each treatment group, blue significance marks indicate the comparison of treatment groups (e.g., 1 mM DTT) to no treatment groups (e.g., 0 mM DTT) for each worm strain, and red significance marks indicate comparison of *metr-1;rips-1* mutant strain to *metr-1* mutant strain for each treatment group

The resistance phenotype depicted in Additional file 1: Fig. S3B shows that no wild-type N2 worms develop from embryos to adults in 4 days in the presence of 5 mM DTT, as compared to a *rips-1* mutant, where almost all become adults. We quantified this survival at 0, 2.5, and 5 mM DTT. Without DTT, 88.5–95% of both worm strains developed into adulthood. At 2.5 mM DTT, 17.7% of the wild-type N2 strain develop into adults, while at 5 mM DTT, no wild-type worms reach adulthood as all developmentally arrested as early larva (Fig. 3a). For the *rips-1* mutants, 97.8% and 88.7% of worms were adults after 4 days at 2.5 and 5 mM DTT, respectively. We found similar levels of resistance for independent *rips-1* alleles (Additional file 1: Fig. S4A and B). Neither bacterial food nor growth media noticeably affect DTT resistance in a *rips-1* mutant (Additional file 1: Fig. S4C). We then tested if the resistance found in our mutants extended to other thiol-reducing agents. In the presence of 2.5 mM β-mercaptoethanol (2-ME), 84% of *rips-1* mutants develop into adults compared with 2.6% of wild-type N2 (Fig. 3b). We conclude that *rips-1* appears to be the sole gene responsible for resistance to thiol-reducing agent toxicity in *C. elegans*.

### RIPS-1 is a methyltransferase conserved in diverse species but not present in vertebrates

To gain more insight into the role of RIPS-1 beyond its presumed biochemical activity as a methyltransferase, we identified proteins related to RIPS-1 and evaluated previous functional studies. Methyltransferases represent a large gene family, with 208 genes identified in humans [17]. BLAST analysis generated multiple hits based on the methyltransferase domain (pfam 13847: corresponding to residues 175–283 of RIPS-1). For our analysis, we therefore required BLAST hits to have ≥ 30% identity over ≥ 90% of the full 365 amino acid residues of RIPS-1 (Additional file 3). Using the taxids described in Additional file 3, this analysis identified related proteins in multiple species of free-living and parasitic nematodes (roundworms such as *C. elegans*), bacteria, mycobacteria, archaea, a single species of fungi (*Arthrobotrys oligospora*), and multiple non-nematode multicellular organisms, such as *Branchiostoma belcheri* (lancelet), *Acanthaster planci *(crown-of-thorns starfish), and *Strongylocentrotus purpuratus* (purple sea urchin). However, these genes remain uncharacterised in these species. We note that the RIPS-1 residues involved in the six amino acid substitution mutations are well conserved in these putative orthologues (Additional file 1: Fig. S5A). Likewise, these residues are also present in the paralogous *C. elegans* proteins (Additional file 1: Fig. S5B). Based on our criteria, we did not find proteins related to RIPS-1 in any plant, insect, platyhelminth (flat worms), annelida (segmented worms), or vertebrate species (Additional file 3). We also searched for remote homologues using HMMER hmmsearch [18] using the proteins aligned in Additional file 1: Fig. S5A as input but found no likely RIPS-1 orthologues in vertebrates. In support of this, OrthoList [19], which identifies the likely human orthologs of *C. elegans* genes using a meta-analysis of four orthology prediction methods, does not show an orthologous match in humans for *C. elegans* RIPS-1. RIPS-1 therefore represents an unusual methyltransferase found in a limited but diverse group of non-vertebrate species.

### Methionine supplementation partially suppresses susceptibility to DTT

We had established two conditions that caused DTT resistance: high levels of vitamin B12 in combination with active METR-1 (methionine synthetase) and loss-of-function mutations in RIPS-1. To test if the mechanism by which loss of RIPS-1 caused DTT resistance was dependent on active METR-1, we constructed a *metr-1*;*rips-1* double mutant and compared the survival of a *rips-1* single mutant with the double mutant on 5 mM DTT (Fig. 3c). The *metr-1*;*rips-1* double mutant showed comparable resistance to the *rips-1* single mutant, with 77% embryos developing to adults in the presence of 5 mM DTT compared with 90% for the *rips-1* single mutant. In contrast, no embryos of *metr-1* single mutant developed into adults on 5 mM DTT (Fig. 2o). As the DTT resistance phenotype of *rips-1* mutant is not suppressed by the loss of *metr-1*, resistance conferred by loss of RIPS-1 does not require active METR-1.

METR-1 utilises vitamin B12 in the conversion of methyl-tetrahydrofolate and homocysteine to tetrahydrofolate and methionine, which is then converted to SAM. It is likely that RIPS-1 then utilises SAM-derived methyl groups to perform its as-yet uncharacterised role in the cytosol (Fig. 1). Therefore, we wondered if tetrahydrofolate and/or methionine might influence the DTT resistance phenotype. We found tetrahydrofolate highly insoluble above 100 μM in our plate supplementation assays and therefore restricted our analysis to methionine. Development of L1 larvae placed on 5 mM DTT OP50 plates in the presence of methionine at 0, 1, and 10 mM was examined (Fig. 3d). Without methionine supplementation, no development to adulthood was observed for wild type (N2) or the *metr-1* single mutant, while at 10 mM methionine, 32% of wild type (N2) and 0% of *metr-1* mutants were adults after 4 days. For the *rips-1* single mutant, the addition of 10 mM methionine only slightly altered the resistance phenotype, while in the *metr-1*;*rips-1* double mutant, a reduction from 99% adults at 0 mM to 78% adults at 10 mM methionine was observed (Fig. 3d). This result shows that in the presence of RIPS-1, 10 mM methionine supplementation results in partial DTT resistance, but when RIPS-1 is absent, methionine supplementation does not enhance DTT resistance. This suggests that both RIPS-1 and methionine may influence resistance to DTT via the same molecular pathway.

### Combined loss of *rips-1* and *mce-1* results in enhanced DTT resistance

Due to the role established for vitamin B12 and METR-1 in DTT resistance, we performed a targeted RNAi modifier screen aiming to identify additional genes that might suppress or enhance DTT sensitivity in a *rips-1* mutant. *C. elegans* genes involved in vitamin B12 processing, the methionine cycle, the folate cycle, the transsulfuration pathway, the AdoCbl processing, the propanoic acid pathway, and the propanoic acid shunt were identified from a review of the literature [20–25]. RNAi clones for the *C. elegans* genes targeted along with pathways and gene functions are listed in Additional file 2: Table S2. RNAi was performed in the *rips-1* mutant strain in the presence and absence of DTT. In the absence of DTT, none of the 24 genes tested resulted in any additional phenotypes in the *rips-1* mutant background in comparison with the wild-type N2 background. Testing of the 24-gene mini-RNAi library in the presence of DTT led to the identification of a strong enhancer of *rips-1* DTT resistance, the methylmalonyl-CoA epimerase gene (*mce-1*) (Fig. 3e). None of the other 23 genes tested reproducibly altered the DTT resistance phenotype. We confirmed the *mce-1* enhancer result by constructing an *mce-1;rips-1* double mutant (Fig. 3e, f). Importantly, the *mce-1* single mutant confers no resistance to 5 mM DTT, being as susceptible as wild-type worms to this reducing agent (Fig. 3f). In combination, however, *rips-1* and *mce-1* mutations result in survival on 7 mM DTT (Fig. 3e, f), compared to a maximum survival concentration of 5 mM for the *rips-1* single mutant (Fig. 3e, f). This result shows that the combined loss of RIPS-1 and MCE-1 confers enhanced resistant to DTT.

### DTT treatment increases *rips-1* transcript abundance

**Fig. 4:**
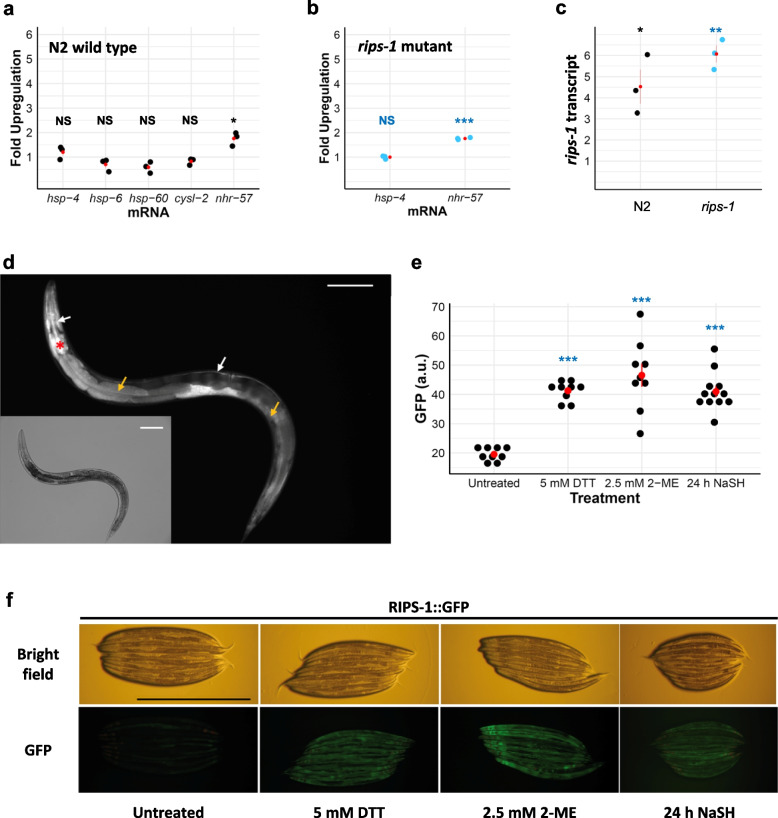
RIPS-1 is induced by thiol-reducing agents and is expressed in the gut and hypoderm. **a**-**c** Assessment of the gene expression of *hsp-4*, *hsp-6*, *hsp-60*, *cysl-2*, and *nhr-57* in N2 wild type (**a**) and *hsp-4 and nhr-57 in a rips-1(ij109)* mutant (**b**) and *rips-1* gene expression in N2 (wild type) and *rips-1(ij109) *(**c**), upon exposure to 5 mM DTT for 24 h was performed via quantitative RT-PCR. All fold changes were normalised to the untreated control of each strain. **d**-**f** A translational RIPS-1::GFP fusion (TP313) (**d**) induced by 5 mM DTT localises to the hypodermis (white arrow) and the gut (yellow arrow) with the *myo-2* transformation marker highlighting the pharynx (red asterisk). GFP quantification (**e**) and bright field with corresponding GFP images (**f**) of worm strain carrying RIPS-1::GFP reporter untreated (most left) or upon treatment with 5 mM DTT (second from left), 2.5 mM 2-ME (second from right), or sodium hydrogen sulfide (NaSH) (most right). Scale bars in **d** and **f** denote 1 mm. In **a**-**c**, and **f**, red points with lines denote the mean and SEM. For **a**-**c**, *p*-values were determined from the *t*-test. For **e**, *p*-values were determined from one-way ANOVA, followed by Dunnett’s test. NS, not significant, *p < 0.05, **p < 0.01, ***p < 0.001. Significance marks indicate the comparison of the treatment group to the untreated control

We next used qRT-PCR to assess the gene expression changes after exposure of synchronised wild-type N2 L4s to 5 mM DTT for 24 h, conditions we found to have a minimal effect on worm development within this time frame. Surprisingly, we found that the expression of markers for the endoplasmic reticulum unfolded protein response (UPR) (*hsp-4*) and mitochondrial UPR (*hsp-6* and *hsp-60*) were essentially unaffected by this acute exposure to DTT (Fig. 4a). We also tested two targets of the transcription factor HIF-1 and found transcript abundance for *cysl-2* to be unaffected whereas the *nhr-57* transcript was increased ~ 2-fold with the addition of DTT (Fig. 4a). *hsp-4* and *nhr-57* transcript levels were also quantified in a *rips-1* mutant where we found similar results to those observed in the wild-type genetic background; *hsp-4* mRNA was unchanged by DTT and an ~ 2-fold increase in *nhr-57* mRNA levels were observed (Fig. 4b).

We then tested if DTT affected the expression of *rips-1* and found that its abundance was increased ~ 4.5-fold in wild-type treated worms compared to untreated controls (Fig. 4c). Therefore, in the presence of DTT, somewhat surprisingly, *C. elegans* strongly upregulates the expression of *rips-1*, the gene that makes them susceptible to DTT. We also measured the *rips-1* transcript levels in a *rips-1* mutant strain and found it was ~ 6-fold upregulated with DTT treatment (Fig. 4c). While mRNA levels may not directly reflect protein presence, this finding argues against a potential simple explanation for DTT resistance in our mutants, whereby *rips-1* mutation results in the loss of DTT uptake. Therefore, *rips-1* and *nhr-57* are upregulated by 5 mM DTT in both wild-type and a *rips-1* mutant strain, but *hsp-4*, *hsp-6*, *hsp-60*, and *cysl-1* are not induced by this treatment.

### RIPS-1 is expressed in the intestinal and hypodermal tissues

To examine the regulation and localisation of RIPS-1, we constructed a GFP protein fusion containing the putative *rips-1* promoter sequence, *rips-1* genomic coding sequence fused at its C-terminus to GFP, followed by *rips-1* 3′UTR region. Due to the proximity and proposed function of neighbouring genes (Additional file 1: Fig. S5C), the 3′UTR was experimentally defined (described in Additional file 2). The resulting plasmid was microinjected into the syncytial gonad of a *rips-1* mutant strain, and transgenic progeny were identified by the expression of GFP and the co-injected transgenic *myo-2*^*prom*^::mCherry pharyngeal marker. Two transgenic strains, TP313 and TP315, were characterised in detail. Both strains carry the transgenic sequences as complex extrachromosomal arrays which are lost naturally in a percentage of their progeny. To ensure that the RIPS-1::GFP fusion transgene was functional, we looked at the survival of transgene-positive and transgene-negative progeny from these strains, reasoning that if the RIPS-1::GFP fusion protein restored RIPS-1 function then it should return sensitivity to DTT in the *rips-1* mutant background. We found no transgene-positive worms developed to L4 or adult stages in the presence of 5 mM DTT with only transgene-negative (*rips-1* mutant) worms surviving these conditions, demonstrating that the transgene restores RIPS-1 function. We next used the transgenic strains to examine the tissue-specific expression of RIPS-1 and found specific transgene expression in the cytoplasm of the gut and hypodermal cells following treatment with 5 mM DTT (Fig. 4d and Additional file 1: Fig. S6b, c and e).

### RIPS-1 is activated by DTT, β-mercaptoethanol, and H_2_S

We next demonstrated that the RIPS-1::GFP transgene responds to DTT treatment as the addition of 5 mM DTT for 24 h induced strong marker expression in the intestine and hypodermal tissues (Fig. 4e, f, and Additional file 1: Fig. S6). Importantly, this verifies our qPCR data, showing that the increase in *rips-1* transcript in response to DTT is also found at the protein level. As *rips-1* mutants are also resistant to 2.5 mM β-mercaptoethanol (2-ME), we then tested the effect of this thiol reductant on the reporter and found similar upregulation of expression to that observed on exposure to DTT (Fig. 4e, f). We also demonstrated the induction of the RIPS-1::GFP marker on DTT with heat-killed *E. coli* OP50-1 and in the absence of bacteria (Additional file 1: Fig. S7). Therefore, as two different thiol reductants upregulate the methyltransferase and as this does not require active growing bacteria, we propose that the effect of DTT on RIPS-1 is unlikely to result from a DTT breakdown product or from bacterial metabolism of the compound. Two RIPS-1 paralogues, R08E5.1 and R08F11.4, have been reported as being upregulated by the transcriptional response to H_2_S [26]. We therefore tested if this regulation extended to RIPS-1 and found exposure to H_2_S for 24 h also results in RIPS-1::GFP marker induction (Fig. 4e, f). RIPS-1 is therefore induced by thiol-reducing agents and H_2_S.

### Hypoxia-inducible factor and mitochondrial dysfunction alter RIPS-1 expression

To identify other conditions and pathways affecting the RIPS-1 expression, we searched the results of published genome-wide expression-profiling data, focussing on studies where *rips-1* was among the final defined gene sets (described in more detail in Discussion). *rips-1* transcript abundance has been reported as significantly downregulated in a SKN-1 gain-of-function mutation that affects mitochondrial function [27, 28], by growth in low selenium [29, 30], and with bacterial food containing high levels of vitamin B12 [31, 32]. *rips-1* transcript abundance was reported as significantly upregulated due to HIF activation [33–36] and mitochondrial dysfunction [37–40]. We therefore used our RIPS-1::GFP marker to determine if the results of these genome-wide transcript abundance studies could be replicated at the protein level in a single-gene experiment.

**Fig. 5:**
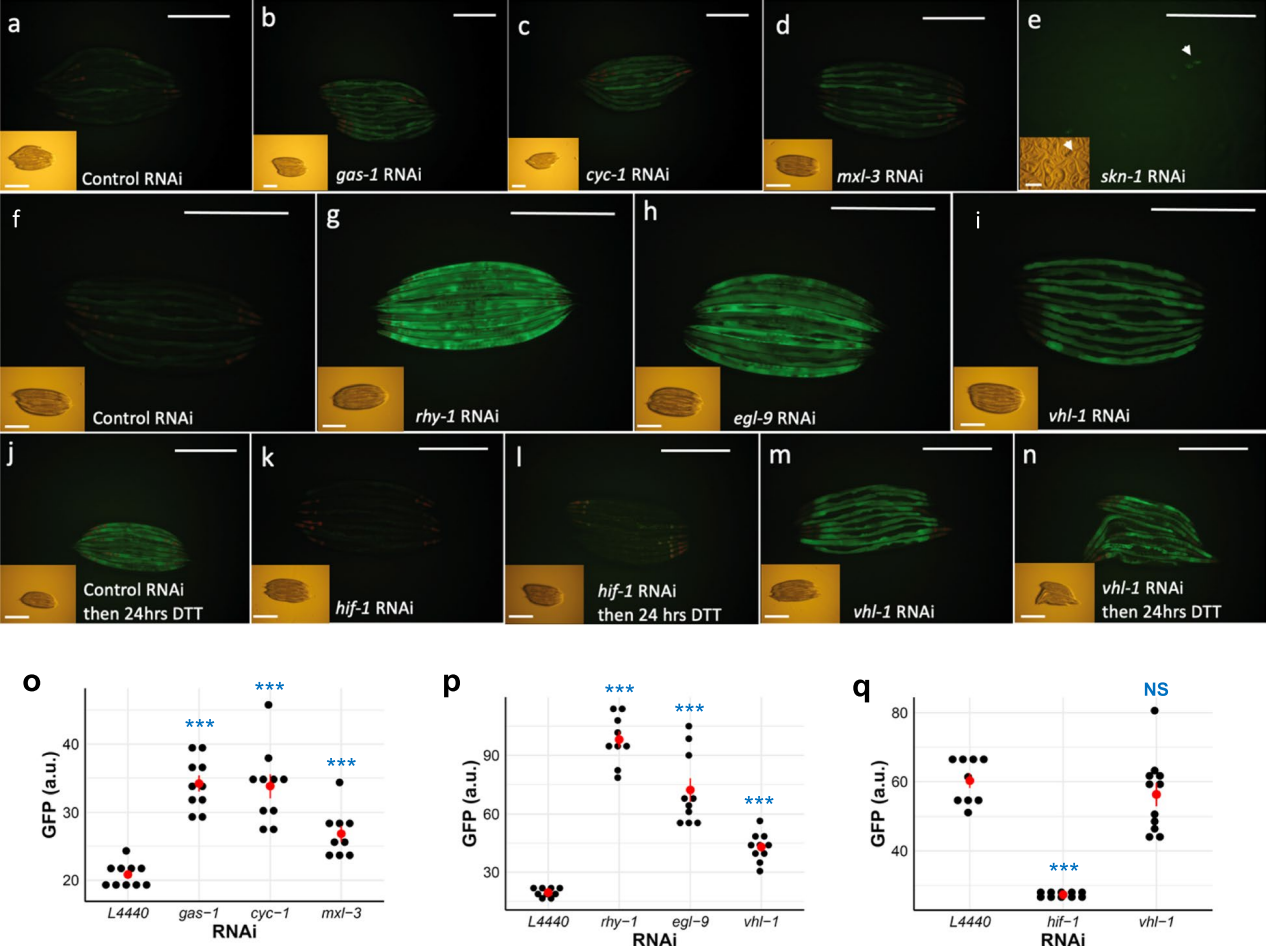
RIPS-1 protein abundance is strongly induced by RNAi of the hypoxia induction pathway genes. Control RNAi causes minimal induction of RIPS-1::GFP (**a**, **f**), and RNAi of the mitochondrial electron transport genes *gas-1* (**b**) and *cyc-1* (**c**) cause mild gut and hypodermal induction, while RNAi of transcription regulators *mxl-3* (**d**) and *skn-*1 (**e**, arrowhead) causes mild gut-specific and embryo-specific induction, respectively. Strong gut and intestine induction were observed following RNAi with the hypoxia pathway-associated genes *rhy-1* (**g**) and *egl-9* (**h**) and induced in a gut-specific manner following *vhl-1* RNAi (**i**). The transcription factor HIF-1 was shown to control RIPS-1 induction, as worms reared on control RNAi with 5 mM DTT treatment showed strong RIPS-1::GFP induction (**j**), while *hif-1* RNAi alone (**k**) or followed by 5 mM DTT treatment (**l**) failed to induce RIPS-1::GFP. The RNAi of hypoxia pathway gene *vhl-1* induced RIPS-1::GFP expression in the gut tissues (**i**, **m**), and this strong induction persisted following 5 mM DTT treatment (**n**). Bright-field images are shown inset; scale bars denote 0.5 mm. GFP quantification for conditions in **a**–**d** is shown in **o**, for **f**–**i** is shown in **p**, and for **j**, **l**, and **n** is shown in **q**. In **o**–**q**, red points with lines denote the mean and SEM, and *p*-values were determined from one-way ANOVA, followed by Dunnett’s test. NS, not significant, ***p < 0.001. Blue significance marks indicate the comparison of the RNAi groups to control RNAi (L4440)

We assessed the effect on our RIPS-1::GFP marker of RNAi of the following genes*: vhl-1*, *rhy-1*, and *egl-9*, which result in the activation of HIF-1; *clk-1, isp-1*, *cyc-1*, and *gas-1*, which affect mitochondrial respiration or ubiquinone production [37, 41], and *spg-7*, a mitochondrial protease required for electron transport chain quality control and mitochondrial ribosome biogenesis [39] (Fig. 5). Mutation of *clk-1 *and* isp-1* [42], RNAi of *cyc-1* [43], and RNAi of *spg-7* [39] have all also been shown to activate mitochondrial UPR. We also included RNAi of *skn-1* and *mxl-3*. As the *rips-1* transcript was downregulated in a *skn-1* gain-of-function mutant [27, 28], we predicted that knockdown of *skn-1* by RNAi might produce the opposite effect and result in RIPS-1::GFP marker activation. RNAi of the transcription factor *mxl-3* was included as deletion of this gene reverses the decreased expression of *rips-1* in the *skn-1* gain-of-function mutant background [27, 28]. We did not test the effect of selenium on the RIPS-1 reporter as its low basal expression makes a reduction in expression difficult to demonstrate.

RNAi of the mitochondria stress marker *clk-1* failed to induce the RIPS-1::GFP marker (Additional file 1: Fig. S8). However, mild induction was observed following RNAi with the mitochondrial stress markers *gas-1* and *cyc-1* (Fig. 5b, c, o). Upregulation of the RIPS-1::GFP marker was also noted in adults following *mxl-3* RNAi and in embryos with *skn-1* RNAi (Fig. 5d, e, o). With the two-generation RNAi feeding protocol used, we found *spg-7* RNAi resulted in a strong developmental arrest phenotype with no noticeable marker induction. In contrast, RNAi of *vhl-1*, *rhy-1*, and *egl-9* produce a striking tissue-specific upregulation of the RIPS-1::GFP marker (Fig. 5g–i, p, and Additional file 1: Fig. S8). As RNAi of *vhl-1*, *rhy-1*, and *egl-9* result in HIF-1 activation, we then tested if upregulation of RIPS-1 by DTT was dependent on HIF-1. We treated the marker strain with RNAi alone for 3 days, then transferred it to RNAi with DTT for 24 h. The empty vector RNAi control and *vhl-1* RNAi both show clear RIPS-1::GFP marker induction on DTT exposure, whereas *hif-1* RNAi strongly suppresses marker induction by DTT (Fig. 5j–n, q and Additional file 1: Fig. S9). These results show that RIPS-1 is mildly induced by mitochondrial stress markers and strongly induced by the hypoxia-inducible factor cascade and controlled by HIF-1.

### The propionate reporter *acdh-1* confirms the dietary impact of B12 deficiency

**Fig. 6:**
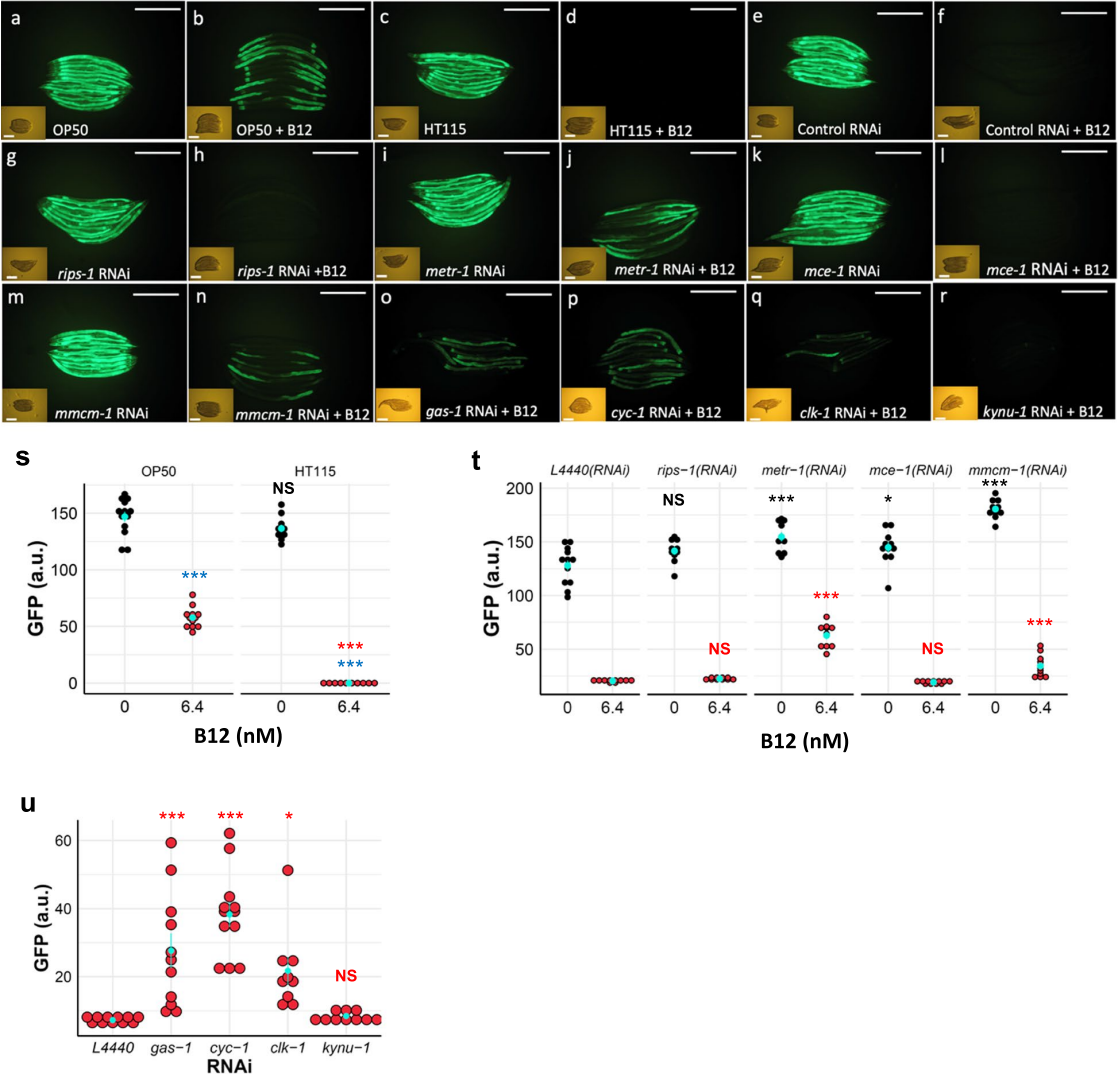
The propionate reporter *acdh-1*^*prom*^::GFP is induced by diet, mitochondrial pathway genes, and vitamin B12 availability. Worms carrying *acdh-1*^*prom*^::GFP (strain VL749) were grown on soy-based NGM agar with 0 nM or 6.4 nM vitamin B12 supplementation. Worms fed on *E. coli* strain OP50-1 strongly induce GFP expression (**a**) that is only partially suppressed by adding B12 (**b**). Worms fed on strain HT115(DE3) also induce GFP expression (**c**), but this induction is completely suppressed by the addition of vitamin B12 (**d**). Worms fed on control RNAi [HT115(DE3) and L4440 empty vector] induce *acdh-1*^*prom*^::GFP expression (**e**), but this induction is suppressed by adding vitamin B12 (**f**). *rips-1* RNAi induces *acdh-1*^*prom*^::GFP (**g**), and induction is suppressed by B12 supplementation (**h**); *metr-1* RNAi induces *acdh-1*^*prom*^::GFP (**i**), and induction is only minimally suppressed by B12 addition (**j**); *mce-1* RNAi induces *acdh-1*^*prom*^::GFP (**k**) and is completely suppressed by the addition of B12 (**l**); *mmcm-1* RNAi induces *acdh-1*^*prom*^::GFP (**m**), and induction is minimally suppressed by B12 addition (**n**). RNAi and supplementation of B12 only minimally suppress the induction by *gas-1* RNAi (**o**), *cyc-1* RNAi (**p**), and *clk-1* RNAi (**q**) but fully suppress the *kynu-1* RNAi induction of this reporter (**r**). Scale bars denote 0.5 mm. Quantification of GFP signals for conditions in **a**–**d** is depicted in **s**, for **e**–**n** is shown in **t**, and for **o**–**r** is shown in **u**. In **s**–**u**, light blue points with lines denote the mean and SEM. *p*-values were determined from one-way ANOVA, followed by Tukey’s (**s**) or Dunnett’s (**t**–**u**) tests. NS, not significant, *p < 0.05, ***p < 0.001. For **s**–**u**, blue significance marks indicate the comparison of the groups treated with vitamin B12 to unsupplemented control, and black and red significance marks indicate the comparison of the RNAi groups to the control (L4440) RNAi groups under no supplement or vitamin B12-supplemented conditions, respectively

When the mitochondrial *mmcm-1* vitamin B12-dependent pathway is disrupted, *acdh-1* is induced with the resulting propionate flux in the mitochondria. An *acdh-1* reporter strain has therefore been found to be an excellent marker for vitamin B12 deficiency [4]. We used this marker strain to examine the effects of peptone media sources, *E. coli* strain food sources, and the effect of mitochondrial gene and B12-dependent pathway gene disruption. The marker remains active when normal animal-based, but particularly soy-based peptone, is used in NGM plates and when OP50-1 is used as the food source (Fig. 6a, s). We did however note that this marker is only mildly repressed by the addition of 6.4 nM vitamin B12 to the NGM plates (Fig. 6b, s), but completely repressed by using *E. coli* HT115(DE3) as a food source in combination with 6.4 nM B12 (compare Fig. 6c, d, quantification in Fig. 6s). As expected, RNAi disruption of B12-dependent genes in the cytosol (*metr-1*) and the mitochondria (*mmcm-1*) also induced the marker strain even in the presence of 6.4 nM B12 (Fig. 6i, j, m, n, quantification in Fig. 6t), but RNAi of neither *rips-1* or *mce-1* affected the induction of this marker in the presence of 6.4 nM B12 (Fig. 6g, h, k, l, quantification in Fig. 6t). The RNAi of genes involved in mitochondrial function, such as *gas-1*, *cyc-1*, and *clk-1* (Fig. 6o–q, quantification in Fig. 6u), induced this marker even in the presence of 6.4 nM B12, whereas activation of marker following RNAi of rhodoquinone biosynthetic enzyme *kynu-1* was repressed by 6.4 nM B12 (Fig. 6r, quantification in Fig. 6u). Thus, dietary vitamin B12 availability and mitochondrial and B12 pathway gene disruptions result in propionate flux in *C. elegans*.

## Discussion

### Novel SAM methyltransferase RIPS-1 is involved in methionine-dependent sensitivity to thiol-reducing agent toxicity

Following several large-scale forward genetic screens in *C. elegans*, a single novel SAM methyltransferase RIPS-1 (R08E5.3) was identified as conferring resistance to the thiol-reducing agent DTT. We found that RNAi knockdown of *rips-1* and the presumed loss-of function stop and splice site mutations behaved identically to the amino acid substitutions, indicating all of the alleles identified for *rips-1* from our mutagenesis screen are loss-of function. It is remarkable that a single methyltransferase enzyme was identified multiple times during separate screens for resistance to thiol-reducing agents, represented by a wide range of distinct mutations; nevertheless, the main function of this novel methyltransferase remains to be established. While this manuscript was in preparation, a second paper confirmed many of the findings we describe, namely that RIPS-1 is the sole gene conferring resistance to DTT, and this resistance is linked to the methionine cycle and vitamin B12 availability [10]. This group’s study also described a link to the IRE-1/XBP-1 unfolded response pathway at very high (10 mM) levels of DTT [10], levels that we found to be lethal to worms in all our assays. This study did not uncover the important link to the HIF-1 signalling cascade which we describe.

It is intriguing to speculate that this protein may play a role in adaptation to low oxygen levels; it is regulated by HIF-1 and RIPS-1 homologues are found in organisms that can adapt to fluctuating O_2_ levels, such as marine invertebrates and nematodes. The RIPS-1 methyltransferase is induced by thiol-reducing agents and H_2_S, with induction potentially resulting in methionine/SAM deficiency that ultimately leads to toxicity; most notably a dramatic developmental delay and ultimately larval lethality (Fig. 1). Both these features can be overcome by supplementing with vitamin B12 or methionine. Cobalamin compounds such as vitamin B12 have been shown to be extremely effective in catalysing the autoxidation of sulfhydryl compounds to disulfides [44, 45]. This reported property of cobalamin could potentially provide a simple explanation for our results, with vitamin B12 essentially deactivating DTT. However, our finding that both vitamin B12 and active METR-1 are required to overcome the effects of DTT on development does not support this mechanism. We observe a strong link to the methionine cycle, and via vitamin B12 dependence the mitochondrial electron transport chain. Bacterial food source influences thiol toxicity in *C. elegans* since diet is the sole source of vitamin B12. Therefore, bacterial strains that make sufficient vitamin B12 and/or media that contains high levels of vitamin B12 allow the survival of nematodes in the presence of strong thiol-reducing agents. Vitamin B12 has previously been reported to play key roles in *C. elegans* development [3], and the availability of this essential vitamin controls the ability of *C. elegans* to break down toxic propionate and imparts metabolic plasticity [4].

### *rips-1* regulation

The SAM methyltransferase *rips-1* was reported as significantly regulated by several groups using genome-wide expression analysis but was not further characterised in these studies. Two publications reported that activation of hypoxia-inducible factor (HIF-1) resulted in a significant increase in *rips-1* transcript abundance. The first of these examined mutants in negative regulators of HIF-1 using RNA-seq to profile gene expression regulated by the hypoxia-response pathway under normoxic conditions. *rips-1* was found to be upregulated in each of the three loss-of-function single mutants *egl-9(sa307)*, *rhy-1(ok1402)*, and *vhl-1(ok161)* compared to wild type [35, 36]. *rips-1* was moderately downregulated in the HIF single mutant, *hif-1(ia4)*, and in an *egl-9;hif-1* double mutant [35, 36]. Similarly, work from the Powell-Coffman Lab [33, 34] used microarrays to profile the expression in *rhy-1(ok1402)* and *egl-9(sa307)* single mutants and a *swan-1(ok267);vhl-1(ok161)* double mutants under normoxia. They defined a set of 219 genes upregulated in each mutant, with *rips-1* upregulated from 9.8- to 14.7-fold. Additionally, the authors provided evidence that *rips-1* could be a direct target of HIF-1 by ChIP-seq [33, 34]. Our results confirm a link between *rips-1*, HIF-1, and the hypoxia induction pathway.

Two studies have indicated that *rips-1* was changed upon induction of mitochondrial stress. The first of these used microarray of *clk-1(qm30), isp-1(qm150)*, and *cyc-1(RNAi)* mutants, which affect mitochondrial respiration or ubiquinone production, to define a set of 73 genes where expression was altered in the same directions in each of the three mutants, with *rips-1* among the most significantly upregulated genes found in the overlap set [37, 38]. The authors showed no effect on lifespan due to the knockdown of *rips-1* by RNAi [37]. Both *clk-1(qm30)* and *isp-1(qm150)* mutations have been shown to activate the mitochondrial UPR marker *hsp-60*^*prom*^::GFP [42], while *cyc-1* (C54G4.8) RNAi activates the *hsp-60*^*prom*^::GFP and *hsp-6*^*prom*^::GFP mitochondrial UPR markers [43]. The second study used microarray analysis to identify a set of 685 genes upregulated due to mitochondrial stress resulting from *spg-7* RNAi [39, 40]. *spg-7* encodes a mitochondrial protease required for electron transport chain quality control and mitochondrial ribosome biogenesis. *rips-1* is induced ~ 7-fold, making it the ~ 60th most induced gene in the set, two of the three *rips-1* paralogs were also described in the upregulated transcript set, *R08F11.4* (induced ~ 43-fold—5th most induced) and *R08E5.1* (induced ~ 2.3 fold—~ 350th most induced) [39, 40].

Profiling the transcriptional response to H_2_S identified the *rips-1* paralog, R08E5.1, in a set of 17 genes significantly changed by exposure to 50 ppm H_2_S for 1 h [26, 46]. Additionally, *R08E5.1* and another *rips-1* paralogue, *R08F11.4*, were in a set of 30 genes with the greatest increase in transcript abundance after 12 h exposure to 50 ppm H_2_S [26, 46]. Although *rips-1* was not found in the study, we reasoned that due to the regulation of its paralogues by H_2_S and the similarities in DTT, β-mercaptoethanol, and H_2_S, *rips-1* may also respond to H_2_S. Exposure of our marker strains to sodium hydrogen sulfide (NaSH) for 24 h significantly increased RIPS-1::GFP marker expression. Changes in transcript abundance to H_2_S were also reported to vary dependent on bacterial diet [26] an observation that could potentially be linked to the availability of vitamin B12.

A set of 87 core diet response genes that change depending on the bacterial food source were defined by microarray expression profiling [31, 32]. *rips-1* shows an almost 3-fold downregulation in young adult worms fed with either *E. coli* HT115 or *Comamonas aquatica* DA1877 compared to *E. coli* OP50. The levels of vitamin B12 available to worms were shown to be much greater in *Comamonas* DA1877 (which synthesises vitamin B12) than in *E. coli* 0P50 diet [6]. Based on the expression of the *acdh-1*^*prom*^::GFP marker, the bacterial strain HT115 is thought to provide moderate levels of B12 to the worms [7, 31].

### Potential role of RIPS-1 in thiol toxicity

A proposed model for the role of RIPS-1 in B12-dependent thiol toxicity is depicted in Fig. 1. Vitamin B12 affects the methionine/SAM pathway having a positive impact on fertility and development; secondly, it lowers levels of propionyl-CoA thereby mitigating the toxicity associated with propionic acid build-up. When vitamin B12 is limiting, it is conceivable that METR-1 and MMCM-1 compete for this cofactor. The mechanism by which the *mce-1*;*rips-1* double mutant enhances resistance to DTT could be due to the resulting increased vitamin B12 availability for METR-1 to make methionine. The addition of DTT increases the abundance of RIPS-1 which in wild types is likely to result in increased RIPS-1 methyltransferase activity that may deplete levels of methionine/SAM and result in sensitivity to DTT. However, when RIPS-1 is mutant, methionine/SAM levels may remain normal allowing survival on DTT. In the absence of METR-1, methionine/SAM level may again be depleted, decreasing survival in DTT.

In nematodes and a limited subset of metazoans including annelids, molluscs, and trematodes, there is an ability to switch from aerobic to anaerobic metabolism by reversing the TCA cycle [47]. This results in reversing the succinate to fumarate conversion by using the unique and highly reduced electron acceptor rhodoquinone. This process also reverses the MMCM-1-dependent cycle thereby removing dependence on adenosyl cobalamin and leads to the production of toxic propionate. It could be hypothesised that one potential function of the HIF-1-induced SAM methyltransferase RIPS-1 is in the biosynthesis of reduced rhodoquinone as an adaptation to anaerobic conditions. Interestingly, RIPS-1 is also listed as containing a ubiquinone domain Ubie_Methyltran (Phenobank; https://worm.mpi-cbg.de/phenobank/cgi-bin/ProteinPage.py?GeneID=511989). The fine balance between this ubiquinone to rhodoquinone switch, reversal of the vitamin B12-dependent TCA cycle, and control of propionate build-up must be carefully regulated and is highly dependent on the redox poise of the cytosol and the mitochondria. A role for mitochondrial activation was also recognised using an RNAi-based approach, where key mitochondrial gene disruption led to the *acdh-1* propionate marker induction, this was significant for *gas-1*, *cyc-1* and to a lesser extent *clk-1*.

## Conclusions

RIPS-1 plays a key role in the response to environmental conditions such as H_2_S, O_2_, methionine availability, and dietary vitamin B12 availability. Intriguingly, this uncharacterised SAM methyltransferase is induced by thiol-reducing agents only for the nematode to become sensitive to the associated toxicity, possibly via the resulting depletion of methionine. This study highlights the important balance between vitamin B12-dependent reactions in the cytosol and mitochondrion and the significant role played by diet in these processes.

## Methods

### Growth and maintenance of *C. elegans*

*C. elegans* strains were maintained as described in [48]; strains used in this study are shown in Additional file 2: Table S3. However, as our DTT assays were found to be sensitive to both bacterial food and growth media (see the “Results” section), NGM to be used in DTT assays was made using soybean peptone (Sigma 70178, peptone from soybean, enzymatic digest) and high purity agar (Sigma 05038, agar for microbiology), and only freshly grown bacterial cultures, grown overnight the day before use, were used to seed NGM-DTT plates. DTT was made by dissolving DL-dithiothreitol (D0632 Sigma) to 1M in water (Sigma W4502, Nuclease-Free Water, for Molecular Biology) with single-use aliquots stored at − 20 °C and used within 1 month. DTT was added to NGM at 55 °C along with the other standard additives [48]. Set plates were dried for 1 h in a flow hood, seeded with fresh *E. coli*, left in a 20 °C incubator in a plastic box with no lid overnight, and then used the following day. Except where noted, the bacterial food used was *E. coli* OP50-1 (from the Caenorhabditis Genetics Center, CGC) which was grown in standard LB broth with Streptomycin (Sigma) from a 12.5-mg/ml stock dissolved in H_2_O, final concentration 12.5 μg/mL. *E. coli* HT115(DE3) (available from the CGC) transformed with an empty vector RNAi was grown in ampicillin at a final concentration of 100 μg/mL. Heat-killed OP50-1 was produced by heating an overnight culture to 60 °C for 30 min, prior to plate seeding.

### Embryo preparations and synchronous cultures

Mixed-stage cultures were grown on NGM-OP50-1 plates until they contained a large population of gravid hermaphrodites and just prior to the food supply being exhausted. Strains were collected, and the embryos were isolated essentially as described [48] but with the following changes; worms were lysed in a total volume of 5 mL using 0.25 mL 5N KOH (final concentration 0.25N) and ~ 0.06–0.5 mL NaOCl (10%, Sigma 1056142500) (volume dependent on the age of the NaOCl solution) and three wash steps each with 12 mL of M9 buffer. To prepare synchronous cultures, the bleached embryos were added to ~ 18 mL M9 buffer in a 90-mm Petri plate and allowed to grow overnight at 20 °C. The resulting starved L1s, which are synchronised at the beginning of the first larval stage, were transferred to 15-mL tubes, washed twice with 12 mL of M9 buffer, added to growth plates using low binding pipette tips, then grown until the required developmental stage.

### Plate supplementation

Vitamin B12 (cobalamin) was purchased in its precursor form cyanocob(III)alamin (CNCbl) (Sigma V2876). CNCbl is converted to the physiologically active forms of vitamin B12, primarily, methylcobalamin (cytoplasmic), and adenosylcobalamin (mitochondrial). CNCbl was prepared as a 10-mM stock in water and used at a concentration of 6.4–64 nM in NGM plates. Methyl THF (Sigma M0132) was added to NGM media during cooling to give a concentration of 100 μM. Hydrogen sulfide (14.3 M, Sigma, 161527) was diluted to 1M immediately before in water (Sigma, W4502) and added to a concentration of 2.5 mM in NGM plates. l-methionine (Sigma M9625 was added at 1 to 20 mM to NGM media during cooling). To assess the effect of survival in various conditions, ~ 75 embryos, prepared by bleaching as above, were added to 6-cm plates and the number that had developed into adults counted 3–4 days later.

### Microscopy and GFP quantification

For high-powered microscopy, live *C. elegans* were transferred to slides with a 2% agarose/0.065% sodium azide pad in 5 μL M9 buffer, and a coverslip was added and sealed with liquid paraffin. Images were captured using an Axioskop 2 microscope (Zeiss) and an AxioCam MRm camera (Zeiss). Low-power group images of worms were taken by picking live worms to a ~ 10-μL 10 mM sodium azide solution on unseeded NGM agar plates. Images were taken using a Canon PowerShot G6 just as the azide pool dried before worm recovery. GFP Fluorescence was quantified using Matlab (R2022a).

### Ethyl methanesulfonate (EMS) mutagenesis and DTT selection

Synchronised L4 larvae were exposed to freshly diluted 50 mM EMS (Sigma M0880) for 4 h at room temperature (ref: “C. elegans: a practical approach”, chapter “Conventional Genetics” by Jonathan Hodgkin, “Protocol 1- EMS Mutagenesis”, pages 252-253) and selected for increased survival NGM-OP50-1 plates with on 5–7.5 mM DTT. A total of approximately 100,000 genomes were mutagenised in three separate screens with each screen being divided into sub-population to identify independent mutants.

### Whole-genome re-sequencing

Strains were prepared for whole-genome re-sequencing and SNP-based mapping [13] by crossing mutant strains with strain CB4856 (Hawaiian) males. As DTT resistant strains are otherwise wild type in morphology and behaviour, crossed progeny were selected by cloning L4 hermaphrodites from the F1 progeny to standard NGM plates and, after egg laying, testing the F1 mother for heterozygosity of a 2940-bp deletion found in the Hawaiian strain (Left flank: AAACCAACGACTCACTAGAGCGCGTATTTT Right flank: TCATCAAATACTTGCATCAACTCCTGAACG [49];) using primers oADW0165/0166/0168. From crossed F1 plates, ~ 200 F2 L4s were cloned onto NGM OP50-1 plates containing 5 mM DTT. Each resistant strain was then put through a second round of identical selection. To reduce the burden of background mutations some of the DTT resistance alleles were outcrossed two times to N2 prior to characterisation and sequencing, see Additional file 2: Table S3.

Next-generation sequencing (NGS) libraries were prepared and sequenced at Glasgow Polyomics (https://www.polyomics.gla.ac.uk/). Briefly, 200 ng genomic DNA, isolated as described in Additional file 2: Methods, was fragmented using a Bioruptor Pico (Diagenode) (5 s on, 90 s off, for 7 cycles), insert size selected for 550 bp, and whole genome libraries constructed using a TruSeq Nano DNA LT Sample Prep Kit (Illumina). Libraries were quantified using a Qubit dsDNA HS kit (Thermo Fisher) with quality assessed using a 2100 Bioanalyzer (Agilent). Two libraries were pooled for sequencing on an Illumina MiSeq using version 3 sequencing reagents with 2 × 300 cycles. NGS data was analysed at the public instance of the open source, web-based platform Galaxy (usegalaxy.org) [50] using the CloudMap [51] “Hawaiian Variant Mapping with WGS Data” and “Variant Calling Workflows”.

### RNAi by bacterial feeding

RNAi was carried out using dsRNA-producing bacteria as a food source. The method was essentially as described [52] but with NGM plates containing 100 μg/ml ampicillin, 1 mM IPTG, plus or minus 5 mM DTT, and using NGM prepared using soybean peptone and high purity agar (as described above). L4s were washed in M9 in a watch glass before being placed on the primary RNAi plates where they were grown for 24 h then transferred to a secondary RNAi plate. The F1 progeny from the second RNAi plates was scored. All bacterial RNAi strains used to generate figures were from the Ahringer lab *C. elegans* RNAi library [15] and were sequenced to confirm identity or purchased from SourceBioscience. In addition to an empty vector negative control RNAi, *dpy-11* (F46E10.9) was included in all RNAi experiments as a positive control.

### RIPS-1::GFP translational fusion

A 3529-bp fragment of *rips-1*, extending from 1078 bp upstream to just downstream of the 3′UTR, as defined by 3′RACE, was amplified from N2 genomic DNA using *Pfu* Ultra II Fusion HS polymerase (Agilent) with primers oADW0256/258. The amplicon was purified using a PCR Purification kit (QIAGEN), A-tailed using GoTaq2 (Promega), and ligated into pCR2.1TOPO (Invitrogen) to generate plasmid pADW0105. A QuikChange XL kit (Agilent) was used for site-directed mutagenesis with plasmid pADW0105 [*rips-1*(+), pCR2.1 TOPO] as template, using primers oADW0272/273 and following the manufacturer’s protocol but with the following adjusted cycling parameters: 95 °C 2 min × 1 cycle; 95 °C 1 min, 60 °C 1 min, 68 °C 19 min × 18 cycles; 68 °C 19 min × 1 cycle. Site-directed mutagenesis removed the stop codon and introduced an *Xma*I restriction site in the sequence and generated plasmid pLBG003. Site-directed mutagenesis results in a change of RIPS-1 C-terminal residues from QN* to QNPG. GFP was amplified from plasmid pPD95.75 (Addgene) using *PfuUltra* II HS DNA Polymerase (Agilent) and primers oADW0274/0275 and cloned into SmaI/CIAP cut pLBG003 to generate plasmid pLBG007 [*rips-1*^*prom*^::RIPS-1::GFP::*rips-1* 3′UTR] (referred to as RIPS-1::GFP). The identity of plasmid inserts was confirmed by restriction enzyme digest and sequencing using vector primers M13Rev(-29) and M13Uni(-21), while the RIPS-1::GFP junctions were sequenced using oADW0274 (GFP F) and oADW0254 (3′RACE primer). Transgenic strains were created following standard microinjection protocols [53] using a Zeiss Axiovert microscope. The DTT resistant strain TP193 [*rips-1(ij109)*] was microinjected with a mix containing 10 ng/mL pLBG007 [RIPS-1::GFP], 10 ng/mL pADW021(3) [myo-2^prom^::mCherry] (pharyngeal marker), and 100 ng/mL 1 kb ladder (Invitrogen). Four transgenic strains carrying extrachromosomal were generated with strains TP313 and TP315 characterised in detail.

### qRT-PCR

Material for expression analysis by qRT-PCR was prepared by treating the wild-type N2 and the DTT resistant strain TP193 (*rips-1*) with 5 mM DTT for 24 h. Cultures were synchronised by bleaching, as described above, and starved L1s plated on standard NGM OP50-1 for 48 h at 20 °C hours until the L4 stage. L4s were washed from plates using M9 buffer, collected in a 15-mL tube then centrifuged at 1150×*g* for 3 min at 20 °C. The worm pellet was re-suspended in 500 μL M9 and distributed to standard NGM OP50-1 plates (seeded the day before with freshly grown OP50-1) containing either no DTT or 5 mM DTT. After 24 h, the young adults were collected by gentle addition of M9 buffer so that embryos remained in OP50-1 bacterial lawn. Tubes were centrifuged as before, washed once with 12 mL M9 buffer, re-centrifuged, the supernatant removed to 1 mL, transferred to 1.5 mL tube using a low binding tip, centrifuged for 3 min at 2000 rpm, the supernatant removed to minimum volume, and worms frozen at − 80 °C. This process was repeated on three separate occasions to generate biological triplicates for qRT-PCR analysis.

Total RNA was isolated (as described in Additional file 2: Methods), and 500 ng of DNAse I-treated total RNA was used in enzyme-plus and enzyme-minus reverse transcriptions using the AffinityScript qPCR cDNA Synthesis Kit (Agilent) with oligo(dT) primer. Reverse-transcription reactions were diluted with (20 μL + 280 μL H_2_O) and qPCR performed using an Agilent Mx3005P qPCR System following the Brilliant III Ultra-Fast SYBR Green QPCR Master Mix (Agilent Technologies) protocol. Tubulin gamma *tbg-1* (F58A4.8) was used as a normalising gene in all cases with some results confirmed using a second normalising gene, actin *act-3* (T04C12.4). All results are from biological triplicate samples and with each PCR in triplicate.

### Experimental design and statistical analysis

The exact sample sizes (*n*) for each experiment are described in the legends of each figure. Three biological replicates were performed for each experiment and sample collection is described in each experimental protocol. In each quantification panels, the mean with SEM is reported. R (version 4.2.0) was used to perform statistical analysis. Fisher’s exact test (Stats base R package, version 3.6.2) was performed to calculate the significance of a condition or treatment in a categorical format. One-way or two-way analysis of variance (ANOVA) (Stats base R package, version 3.6.2), followed by the post hoc Tukey’s (PMCMRplus package version 1.9.6) or Dunnett’s (DescTools package version 0.99.45) test, was performed to calculate the significance of a condition or treatment when there are three or more groups in the experiments. A two-sided Student’s *t*-test was performed to calculate the significance of a condition or treatment when comparing only two groups. Each statistical test used for each figure panel is described in the legends. All statistical tests results were indicated in each figure as follows: NS, not significant, *p < 0.05, **p < 0.01, and ***p < 0.001.

## Supplementary Information

**Additional file 1: Figure S1.** B12 supplementation alleviates DTT toxicity in a *mmcm-1* mutant but not *metr-1* mutant. Embryos of (A-C) wild type (N2), (D-F) *metr-1(ok521)*, (G-I) *mmcm-1(ok1637)*, and (J-L) *rips-1(ij109)* were added to plates supplemented with 0 (top row) or 5 mM DTT (middle and bottom rows), in the presence (bottom row) or absence (top and middle rows) of 64 nM vitamin B12. Development to adult stage was assessed 4 days later and representative images are shown in panels (A-L). Scale bars denote 1 mm. The number of animals used in this experiment are as follows: (A) (*n* = 118), (B) (*n* = 136), (C) (*n* = 73); (D) (*n* = 197), (E) (*n* = 147), (F) (*n* = 197); (G) (*n* = 59), (H) (*n* = 93), (I) (*n* = 56); (J) (*n* = 109), (K) (*n* = 62), (L) (*n* = 94). (M) Plotted development to adult stage in percentage under the above treatments. *p*-values were determined from Fisher’s exact test. NS not significant, *** *p* < 0.001. For all panels, purple significance marks indicate comparison of mutant worm strains to N2 wild type for each treatment group and blue significance marks indicate comparison of treatment groups (i.e., DTT or DTT+B12) to no DTT groups for each worm strain. **Figure S2.** DTT resistance mutants map to a single SAM methyltransferase gene. (A-B) HA mapping output from DTT resistance screen for (A) *rips-1* allele *ij109* (strain TP193) and (B) *rips-1* allele *ka14* (strain TP251). Clear peak visible on Chromosome V. (C) Protein sequence of RIPS-1 SAM methyltransferase highlighting location of mutation generated via EMS DTT resistance screen. Underlined residues exon/exon junctions and position and nature of mutation highlighted in colour and allele designation in brackets. Location of mutation relative to methyltransferase domain highlighted in cartoon (Pfam (PF13847) Methyltransf_31 residues 176-285; InterPro domain (IPR025714) Methyltranfer_dom residues 176-285. **Figure S3.** The loss of *rips-1* causes DTT resistance phenotype. (A) High degree of identity between *rips-1* (R08E5.3) and its closest homologue R08E5.1 that will account for potential RNAi cross-reaction. (B) N2 wild type [a], *rips-1(ij109)* mutant [TP193] [b], or wild type worms fed on *E. coli* expressing RNAi targeting *rips-1* (*R08E5.3*) [c] or its homologues (*R08E5.1* [d], *R08F11.4* [e], or *K12D9.1* [f]) was treated with 5 mM DTT for confirmation of causative gene for DTT resistance phenotype. Worms harbouring *rips-1(ij109)* allele or fed on *rips-1* RNAi or *R08E5.1* RNAi survived and reached adult stage on DTT while wild type (N2) or worms fed on *R08F11.4* RNAi or *K12D9.1* RNAi arrested development on DTT. Scale bars denote 1 mm. (C) The *rips-1* paralogue R08E5.1 is induced by 5 mM DTT in wild type (N2) as shown via quantitative PCR. Red points with lines denote the mean and SEM. The level of R08E5.1 in wild type increases 4-fold upon exposure to 5 mM DTT (mean 4.527, Standard deviation 1.389). **Figure S4.** Media composition and bacterial food source do not influence DTT resistance of *rips-1* mutant strain. (A-B) Counts for additional alleles of *rips-1*: (A) TP276 [*rips-1(ka23)*] and (B) TP251 [*rips-1(ka14)*]. Survival to adulthood after 4 days on 5 mM DTT treatment was compared to wild type N2. Red points with lines denote the mean and SEM. *p*-values were determined from Student’s *t*-test. *** *p* < 0.001. Significance marks indicate comparison of *rips-1* mutants to wild type. (C) *rips-1(ij109)* embryos were added onto (top row) animal-based peptone NGM or (bottom row) soy plant-based peptone NGM with either (half left) B12-poor *E. coli* strain OP50 or (half right) B12-rich *E. coli* strain HT115, with or without 5 mM DTT. Worms were then viewed as adults after 4 days at 22°C. All growth conditions resulted in development to healthy adult populations. Scale bars denote 1 mm. **Figure S5.** R08E5.3 is a methyltransferase conserved in diverse species. (A) Top BLAST hits from R08E5.3 amino acid sequence (Fig. S2C) as representative sequences: archaea [*Nitrosopumilus maritimus*, WP_012215840.1], mycobacterium [*Mycobacterium mantenii*, WP_083099804.1], bacterium [*Desulfovibrio brasiliensis*, WP_054652021.1], non-nematode multi-cellular eukaryote [*Branchiostoma belcheri*, XP_019632822.1], *Caenorhabditis elegans rips-1* [NP_504045.1], non-Caenorhabditis nematode [*Angiostrongylus cantonensis*, KAE9416730.1], and fungi [*Arthrobotrys oligospora*, KAF3112035.1]. (B) Conservation of amino acids between *rips-1* and its orthologues: *rips-1* [NP_504045.1], R08E5.1 [NP_504044.3], R08F11.4 [NP_504052.1] and K12D9.1 [NP_503823.2]. In (A-B), conserved mutations found in *rips-1* DDT resistance mutants are shown in red above the alignments. (C) Genomic location and genes surrounding *rips-1* (R08E5.3). **Figure S6.** RIPS-1::GFP reporter transgene shows subcellular induction following 5 mM DTT exposure. RIPS-1::GFP transgenic strain (A-C) TP313 and (D-F) TP315 show (A, D) weak gut induction in the absence of DTT (white asterisk in (A) denotes the transgenic pharyngeal marker), but (B-C, E-F) strong induction in gut (arrowed) and the hypodermis (arrowhead) following 5 mM DTT exposure. Scale bars denote 0.1 mm. **Figure S7.** RIPS-1::GFP reporter is induced by DTT in the presence of heat-killed OP50 and in the complete absence of bacteria. Transgenic RIPS-1::GFP (TP313) nematodes were picked to unseeded plates for 1 hour then transferred to plates in the (A) absence or (B-C) presence of 5 mM DTT, with no bacteria (A-B) or (C) heat-killed bacteria (60°C for 30 min), and grown for 24 hours. Insets represent bright field image. Scale bars denote 0.5 mm. (D) GFP quantification of (A-C). Red points with lines denote the mean and SEM. *p*-values were determined from one-way ANOVA, followed by Tukey’s test. *** *p* < 0.001. Significance marks indicate comparison of DTT-treated groups to untreated control. **Figure S8.** RNAi of hypoxia pathway genes induce RIPS-1::GFP reporter expression in TP315. An independent RIPS-1::GFP reporter strain (TP315) was used to carry out the same experiment depicted in Fig. 5. RIPS-1::GFP reporter strain was reared on *E. coli* expressing (A) Control (L4440) RNAi, (B) *rhy-1* RNAi, (C) *egl-9* RNAi, (D) *vhl-1* RNAi, (E) *mxl-3* RNAi, or (F) *clk-1* RNAi. Knockdowns of *rhy-1* and *egl-9* induce RIPS-1::GFP expression in the gut and hypodermis, *vhl-1* RNAi induces RIPS-1::GFP expression only in the gut, while control, *mxl-3*, and *clk-1* RNAi do not cause any induction. Insets represent bright field image. Scale bars denote 0.5 mm. (G) Quantification of RIPS-1::GFP expression in panels (A-F). Red points with lines denote the mean and SEM. *p*-values were determined from one-way ANOVA, followed by Dunnett’s test. *** *p* < 0.001. Significance marks indicate comparison of DTT-treated groups to untreated control. **Figure S9.** Hypoxia induction factor HIF-1 controls RIPS-1 activation on DTT exposure. An independent RIPS-1::GFP reporter strain (TP315) was used to carry out the same experiment depicted in Fig. 5. (A-B) Control, (C-D) *hif-1*, and (E-F) *vhl-1* RNAi feeding were carried out on RIPS-1::GFP reporter strain for 3 days. L4 animals with positive *myo-2* transgenic marker (red pharynx) were then picked onto the corresponding RNAi plates that (B, D, F) were supplemented with 5 mM DTT or (A, C, E) with no DTT for 24 hours prior to imaging. (A-B) Worms reared on control RNAi only showed strong RIPS-1::GFP induction upon treatment with 5 mM DTT, while (C) *hif-1* RNAi alone or (D) followed by 5 mM DTT treatment failed to induce RIPS-1::GFP. (E-F) RNAi of *vhl-1* induced RIPS-1::GFP expression in the gut tissues that persisted following DTT exposure. Insets represent bright field image. Scale bars denote 0.5 mm. Quantification of GFP signals for panels (A-F) is depicted in (G), where red points with lines denote the mean and SEM. *p*-values were determined from one-way ANOVA, followed by Tukey’s *post-hoc* test. NS not significant, * *p* < 0.05, *** *p* < 0.001. In panel (G), blue significance marks indicate comparison of groups treated with 5 mM DTT to untreated control, black and red significance marks indicate comparison of RNAi groups to control (L4440) RNAi groups within no DTT or DTT-treated conditions, respectively.

**Additional file 2: Additional Materials and Methods.** gDNA isolation, Sanger sequencing confirmations of *rips-1* alleles in DTT strains, total RNA extraction, 3′RACE for *rips-1*, and genotyping. **Additional Results.** 3′ untranslated region from *rips-1 (R08E5.3)*. **Table S1.** DTT resistance alleles. **Table S2.** RNAi mini-screen for B12-related pathways. **Table S3.***C. elegans* strains. **Table S4.** Oligonucleotide primers. **Table S5.** Plasmids. Additional References.

**Additional file 3.** Detailed BLAST analysis of RIPS-1.

**Additional file 4.** Raw data for Figs. 2o, 3a, b, c, d, f, 4a, b, d, 5o, p, q, 6s, t, u, and Additional file 1: Figs. S1M, S3, S4A-B, S7, S8, and S9.

**Additional file 5.** Statistical analysis results for Figs. 2o, 3a, b, c, d, f, 4a, b, d, 5o, p, q, 6s, t, u, and Additional file 1: Figs. S1M, S4A-B, S7, S8, and S9.

## Data Availability

The datasets supporting the conclusions of this article are included within the article and its additional files. The sequence data supporting the conclusions of this article are available in the NCBI repository under BioProject PRJNA861730 [54].
