# Supplementary material for: Dietary-derived vitamin B12 protects Caenorhabditis elegans from thiol-reducing agents
Source: BMC Biol. 2022 Oct 8;20:228. doi: 10.1186/s12915-022-01415-y (PMC9548181; doi:10.1186/s12915-022-01415-y)
Supplement: Supplementary file 1 — Additional file 1: Figure S1. B12 supplementation alleviates DTT toxicity in a mmcm-1 mutant but not metr-1 mutant. Embryos of (A-C) wild type (N2), (D-F) metr-1(ok521), (G-I) mmcm-1(ok1637), and (J-L) rips-1(ij109) were added to plates supplemented with 0 (top row) or 5 mM DTT (middle and bottom rows), in the presence (bottom row) or absence (top and middle rows) of 64 nM vitamin B12. Development to adult stage was assessed 4 days later and representative images are shown in panels (A-L). Scale bars denote 1 mm. The number of animals used in this experiment are as follows: (A) (n = 118), (B) (n = 136), (C) (n = 73); (D) (n = 197), (E) (n = 147), (F) (n = 197); (G) (n = 59), (H) (n = 93), (I) (n = 56); (J) (n = 109), (K) (n = 62), (L) (n = 94). (M) Plotted development to adult stage in percentage under the above treatments. p-values were determined from Fisher’s exact test. NS not significant, *** p < 0.001. For all panels, purple significance marks indicate comparison of mutant worm strains to N2 wild type for each treatment group and blue significance marks indicate comparison of treatment groups (i.e., DTT or DTT+B12) to no DTT groups for each worm strain. Figure S2. DTT resistance mutants map to a single SAM methyltransferase gene. (A-B) HA mapping output from DTT resistance screen for (A) rips-1 allele ij109 (strain TP193) and (B) rips-1 allele ka14 (strain TP251). Clear peak visible on Chromosome V. (C) Protein sequence of RIPS-1 SAM methyltransferase highlighting location of mutation generated via EMS DTT resistance screen. Underlined residues exon/exon junctions and position and nature of mutation highlighted in colour and allele designation in brackets. Location of mutation relative to methyltransferase domain highlighted in cartoon (Pfam (PF13847) Methyltransf_31 residues 176-285; InterPro domain (IPR025714) Methyltranfer_dom residues 176-285. Figure S3. The loss of rips-1 causes DTT resistance phenotype. (A) High degree of identity between rips-1 (R08E5.3 [file 12915_2022_1415_MOESM1_ESM.pptx]

## Slide 1
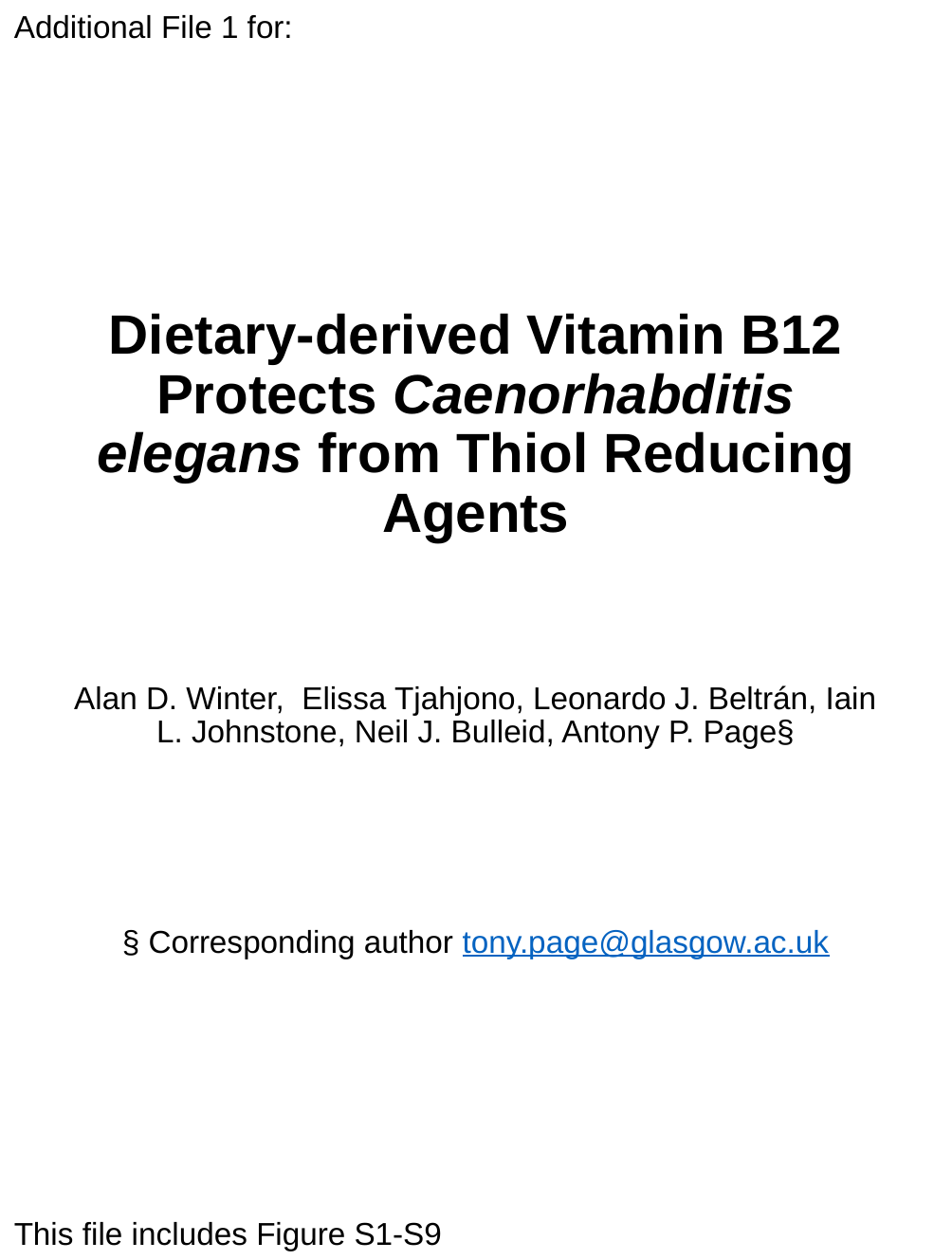

Additional File 1 for:
# Dietary-derived Vitamin B12 Protects Caenorhabditis elegans from Thiol Reducing Agents
Alan D. Winter, Elissa Tjahjono, Leonardo J. Beltrán, Iain L. Johnstone, Neil J. Bulleid, Antony P. Page§
§ Corresponding author tony.page@glasgow.ac.uk
This file includes Figure S1-S9

## Slide 2
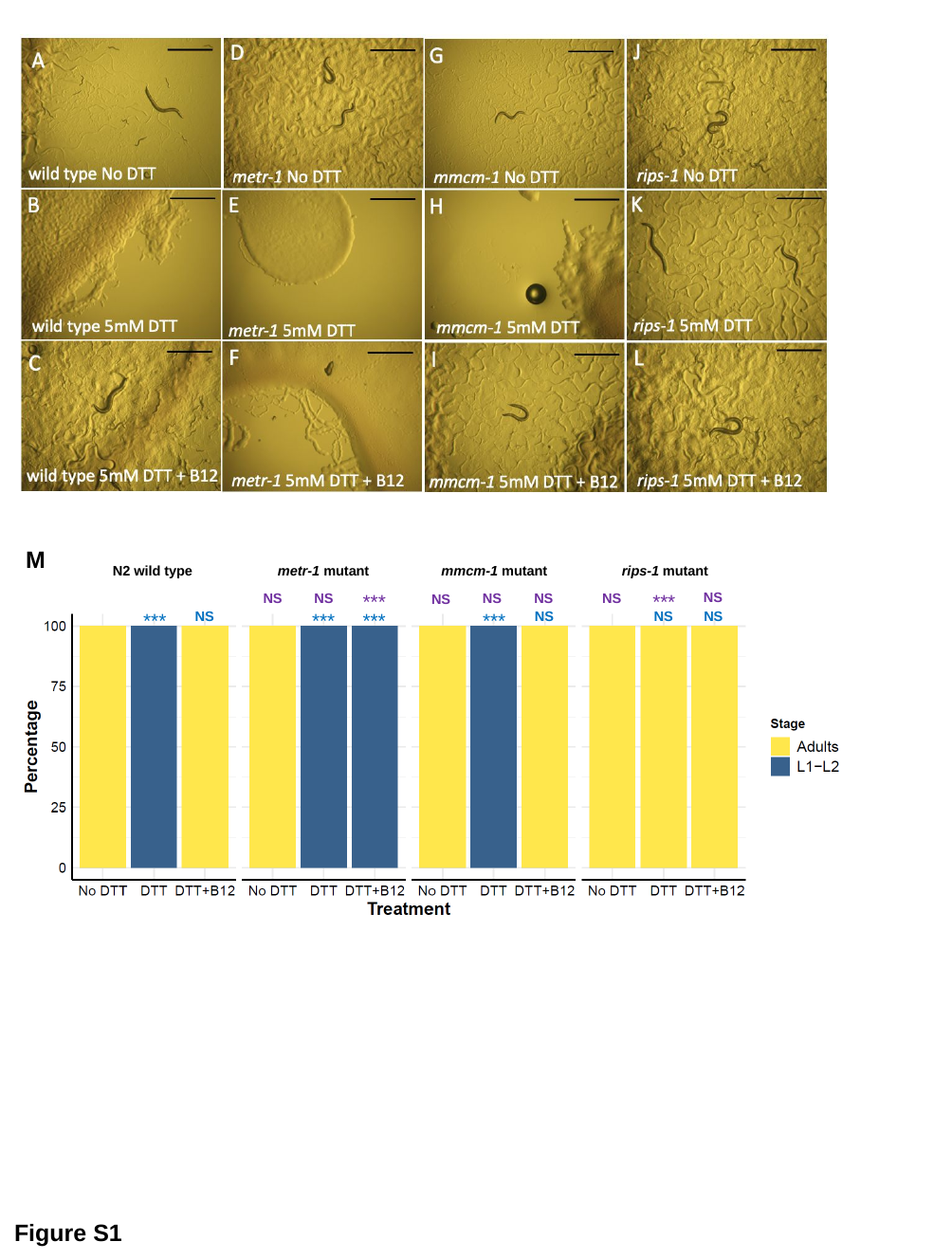

M
rips-1 mutant
N2 wild type
metr-1 mutant
mmcm-1 mutant
NS
NS
NS
NS
NS
NS
NS
***
***
NS
NS
NS
NS
***
***
***
***
Figure S1

## Slide 3
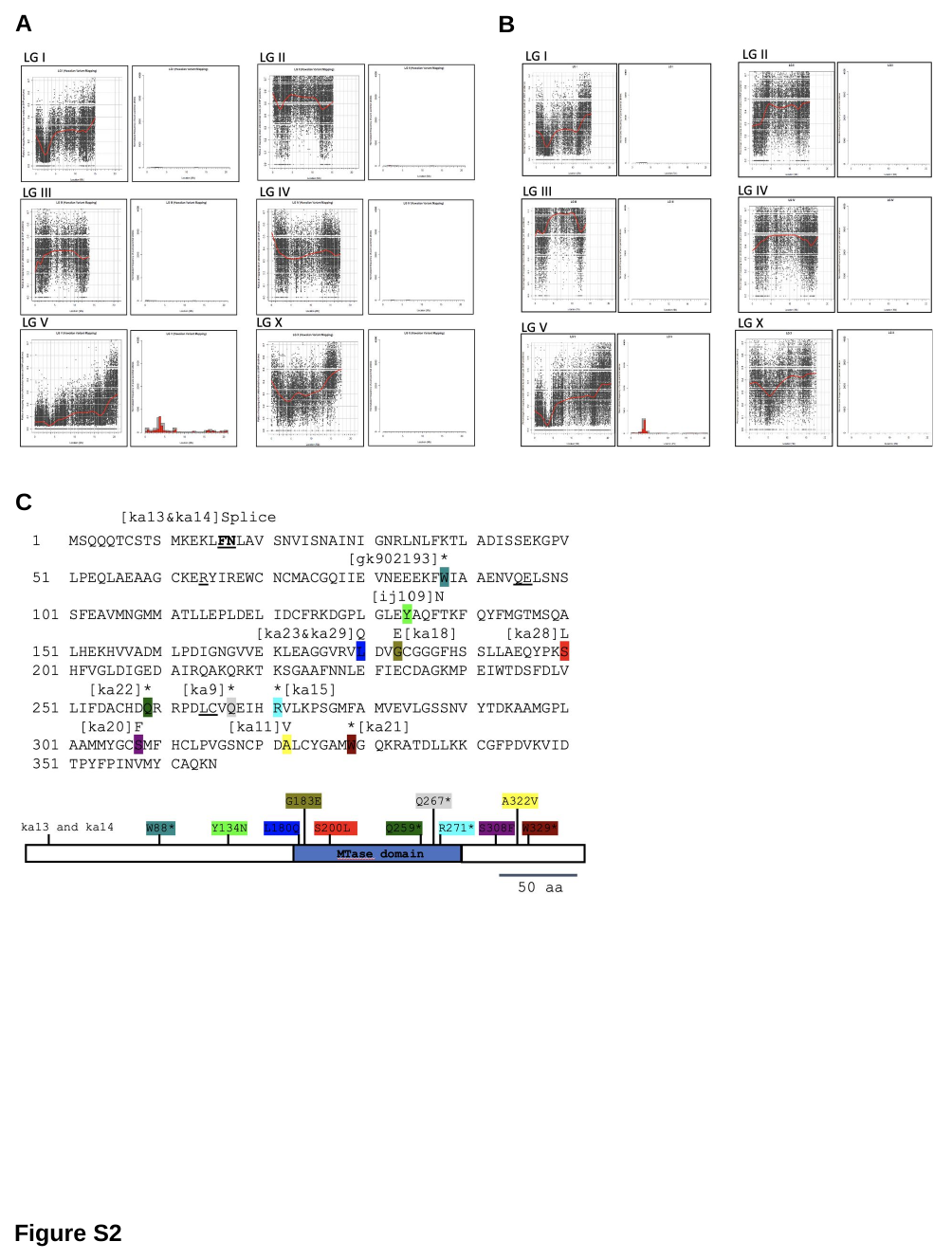

A
B
C
# Figure S2

## Slide 4
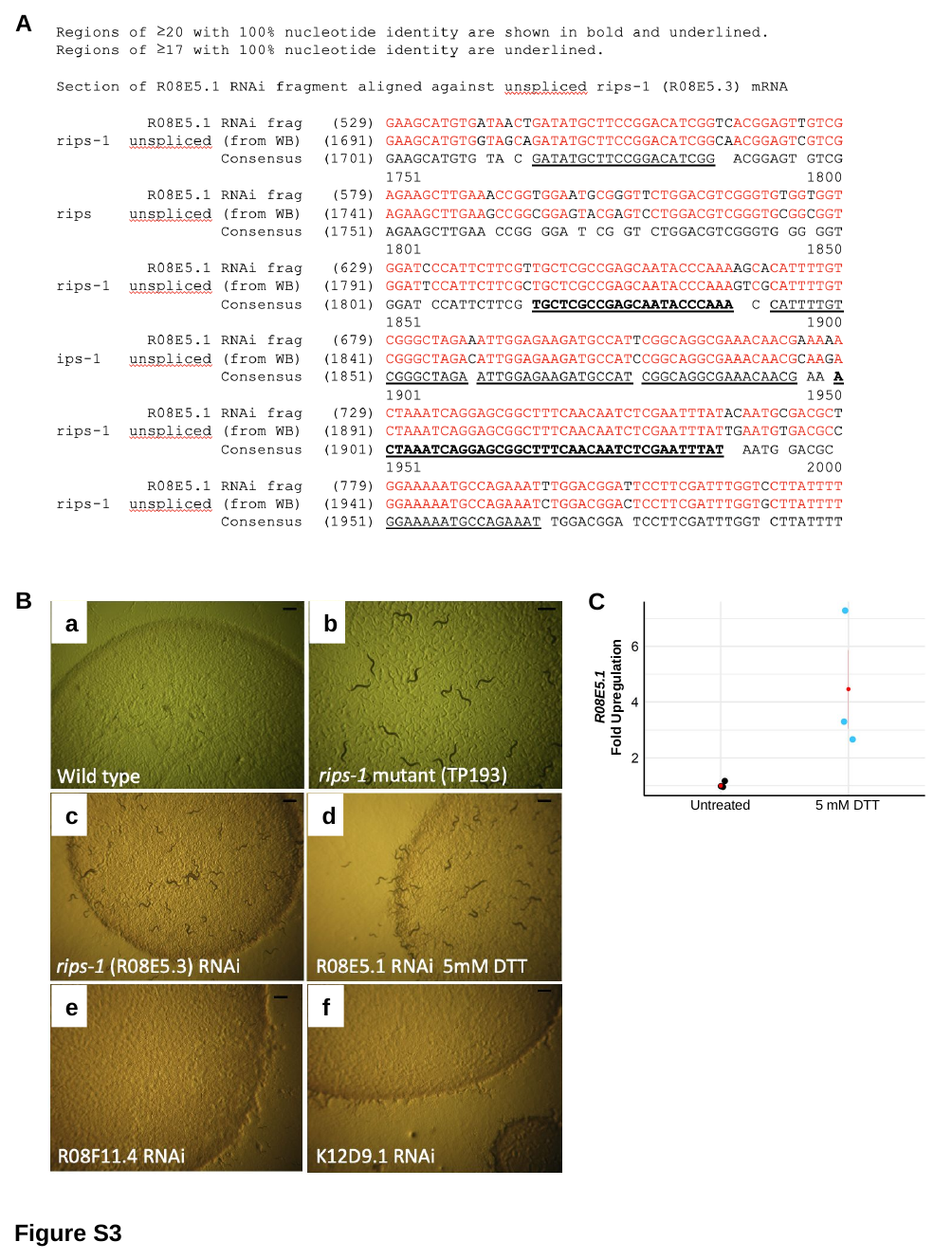

A
B
C
a
b
c
d
f
e
R08E5.1
Fold Upregulation
Untreated
5 mM DTT
# Figure S3

## Slide 5
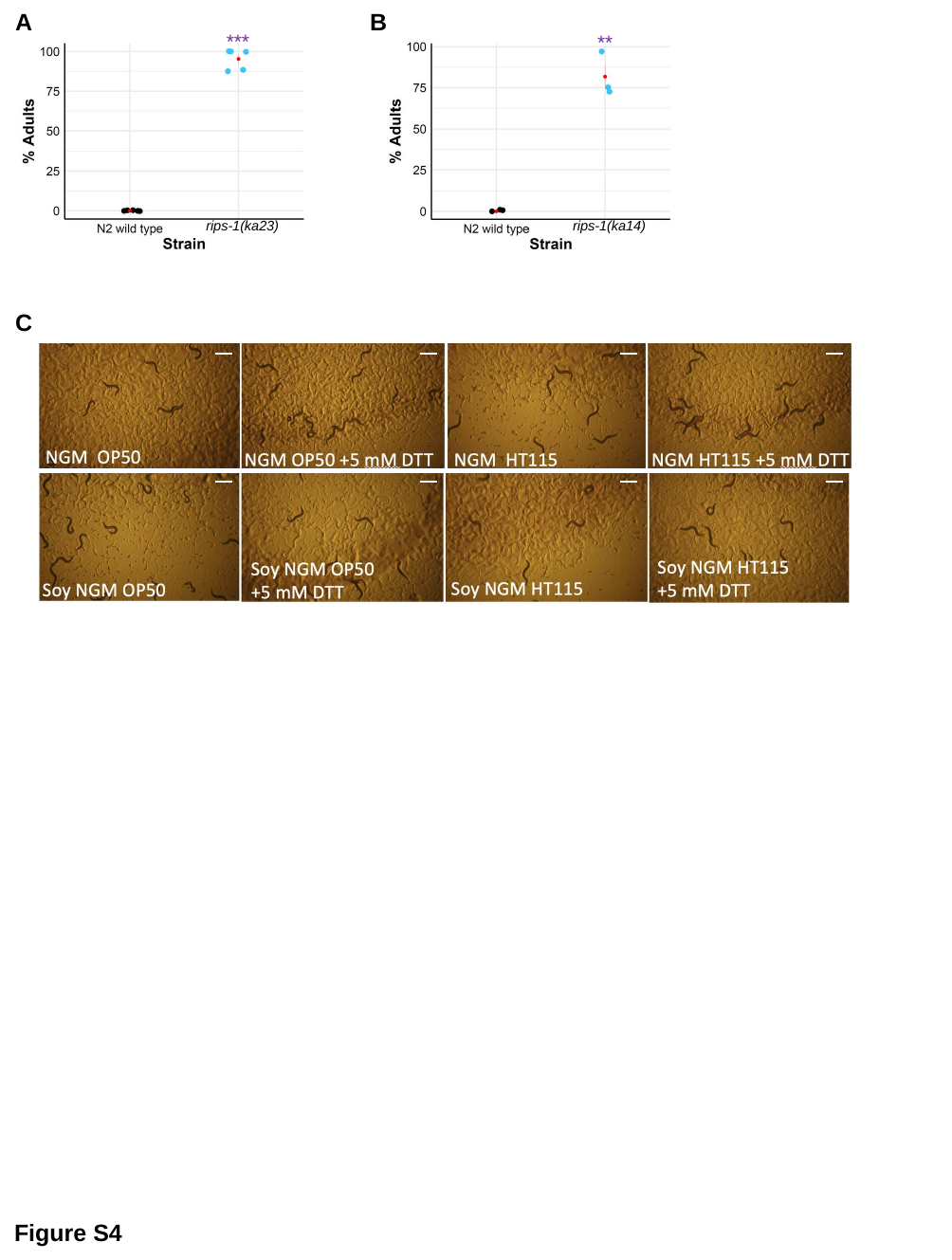

B
A
***
rips-1(ka23)
**
rips-1(ka14)
C
# Figure S4

## Slide 6
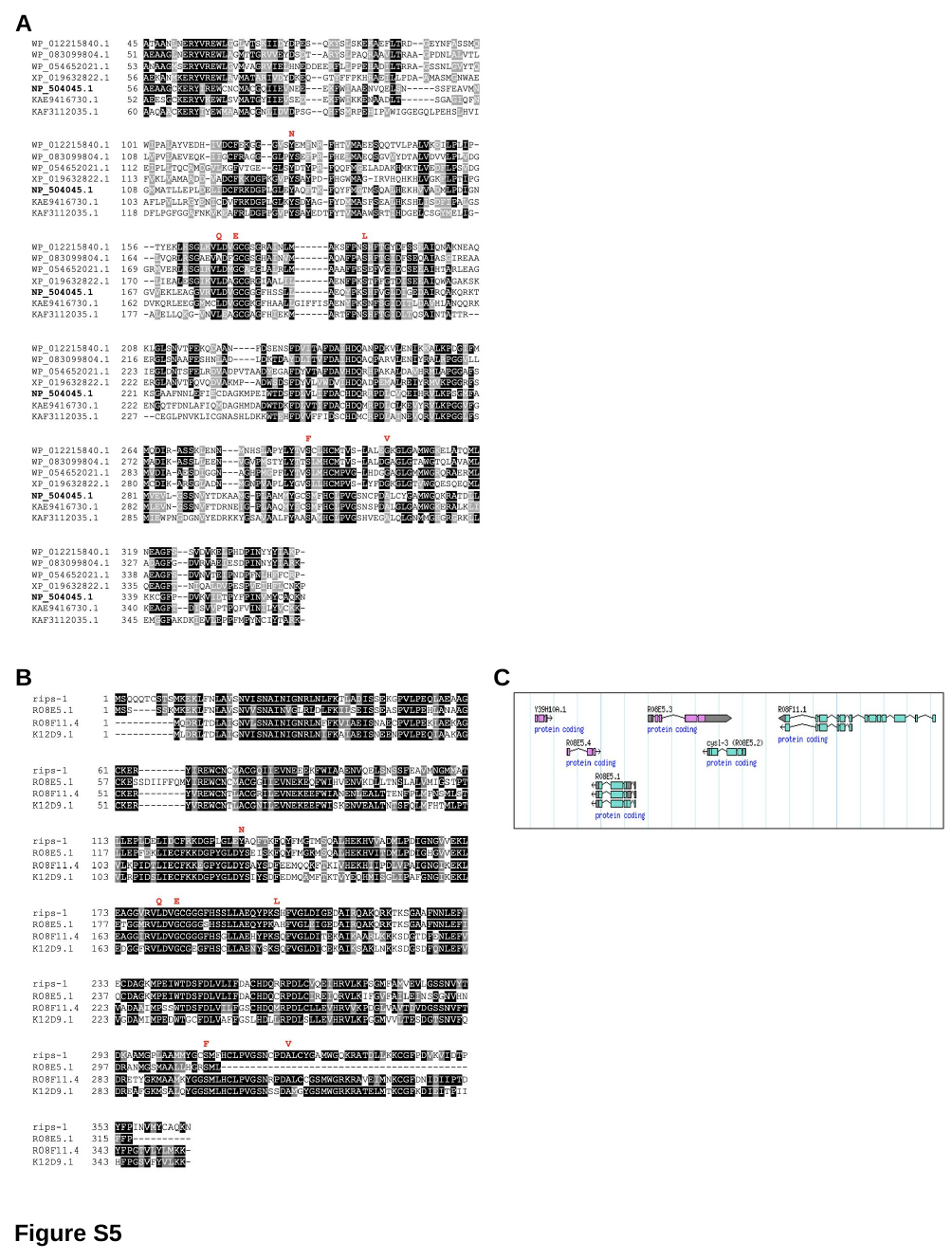

A
B
C
# Figure S5

## Slide 7
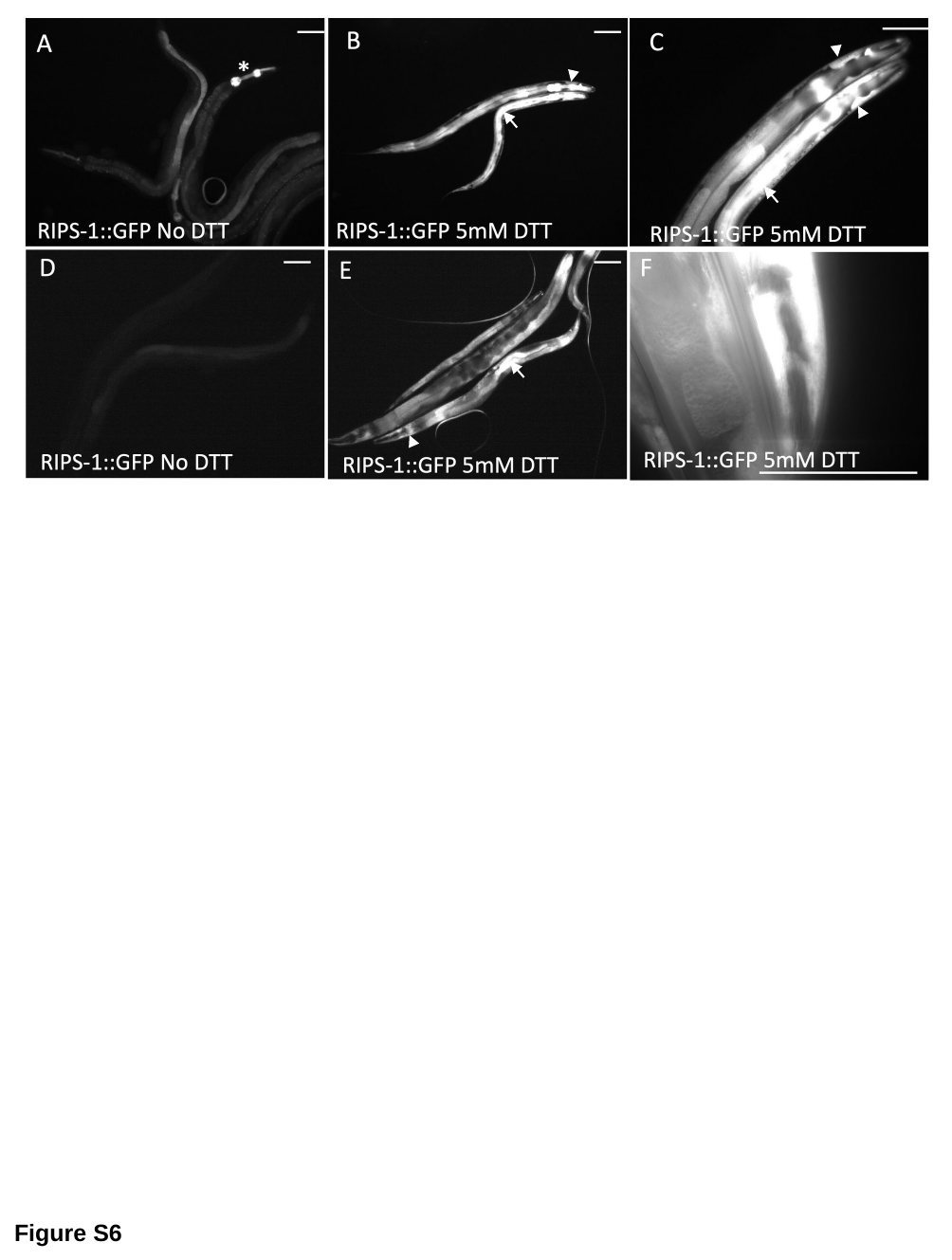

# Figure S6

## Slide 8
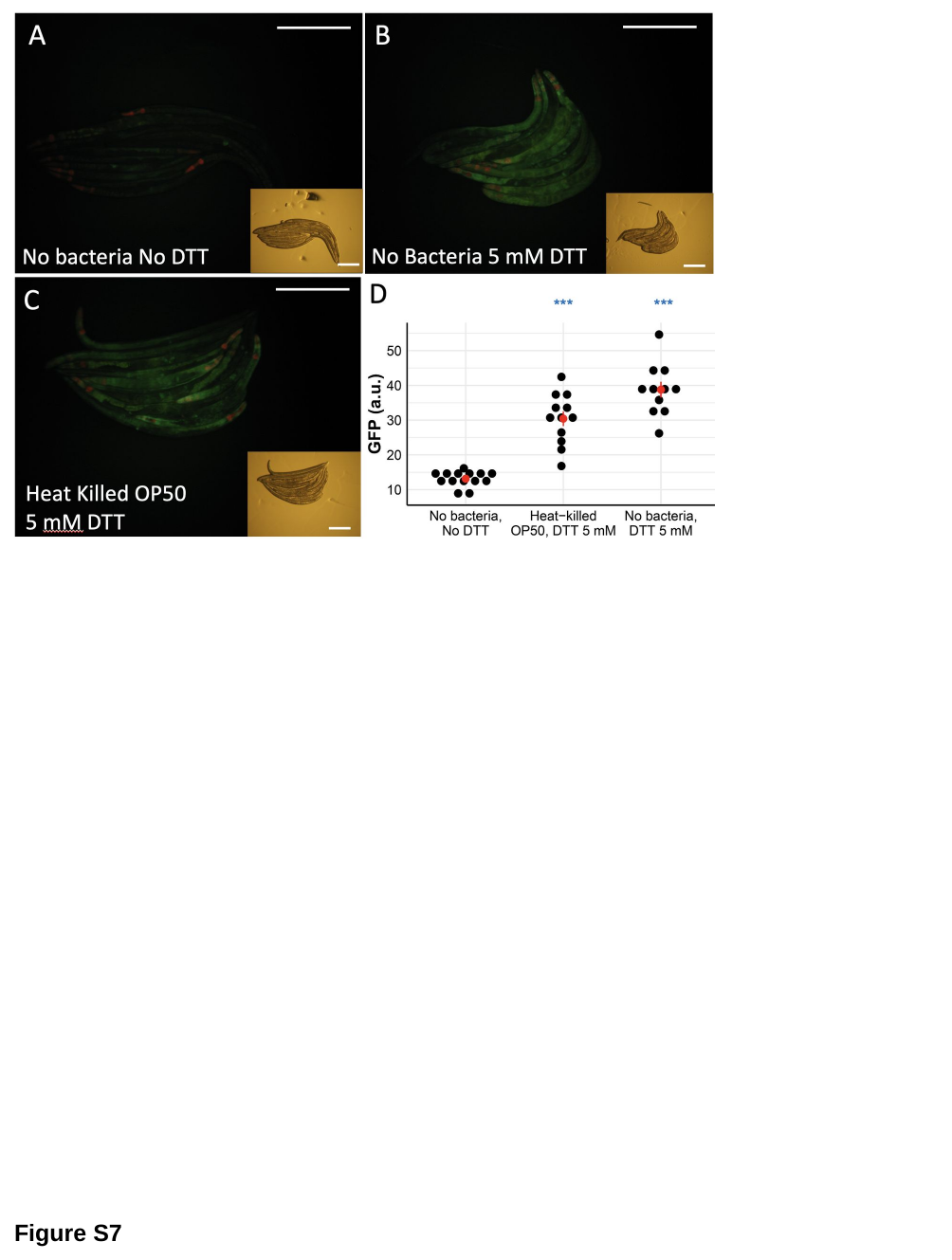

# Figure S7

## Slide 9
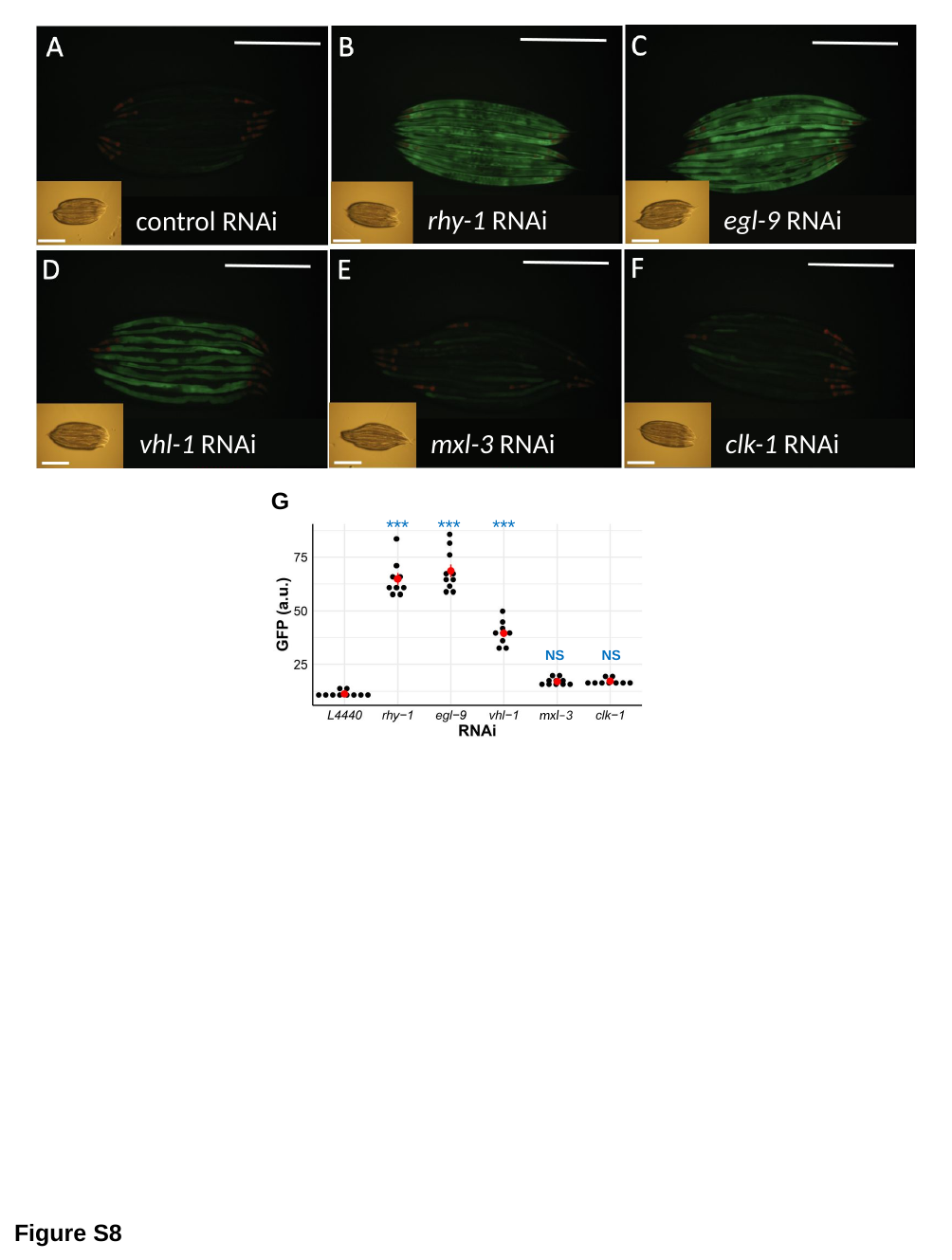

rhy-1 RNAi
egl-9 RNAi
control RNAi
mxl-3 RNAi
clk-1 RNAi
vhl-1 RNAi
G
***
***
***
NS
NS
mxl–3
Figure S8

## Slide 10
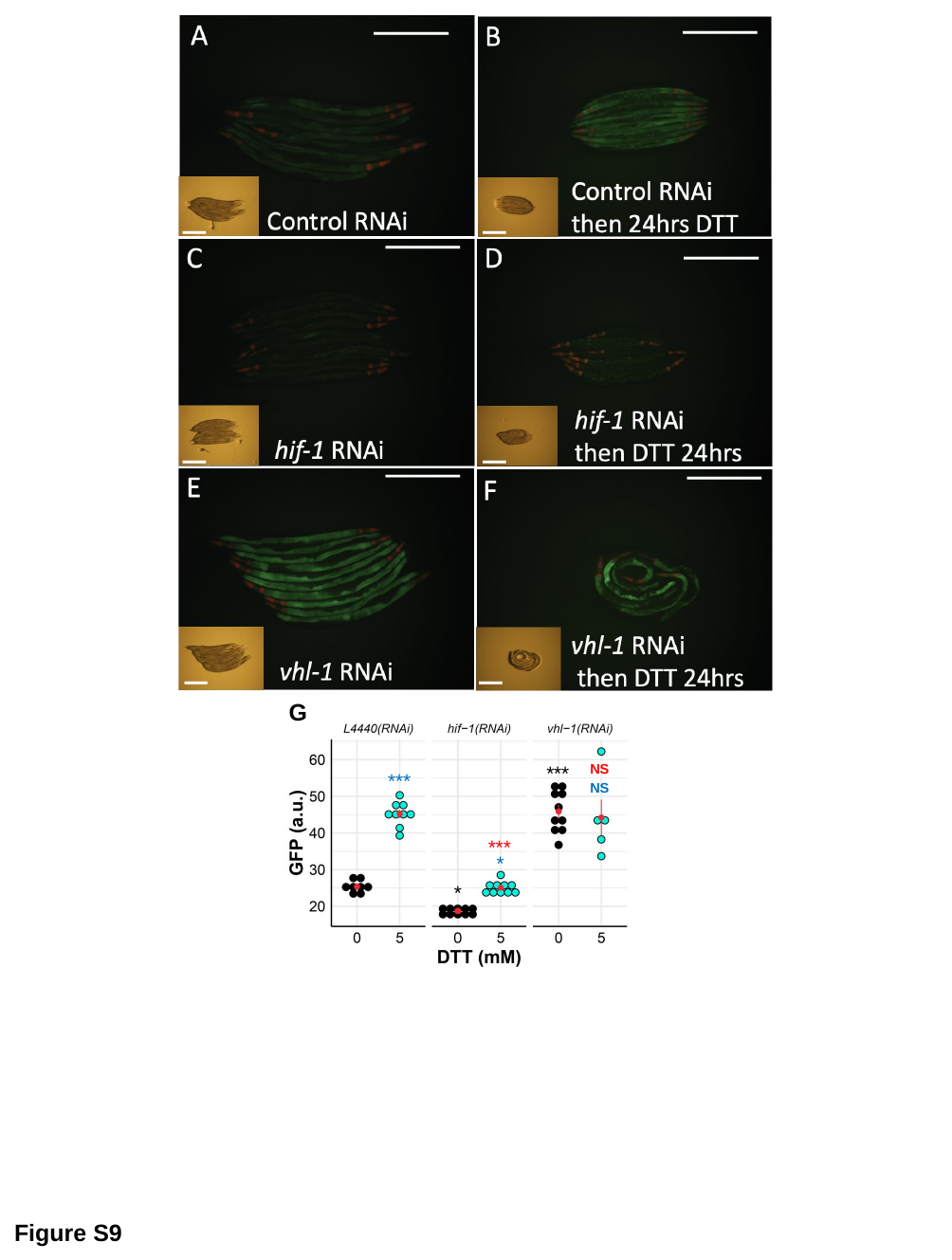

G
NS
***
***
NS
***
*
*
# Figure S9
