# Supplementary material for: Dietary-derived vitamin B12 protects Caenorhabditis elegans from thiol-reducing agents
Source: BMC Biol. 2022 Oct 8;20:228. doi: 10.1186/s12915-022-01415-y (PMC9548181; doi:10.1186/s12915-022-01415-y)
Supplement: Supplementary file 2 — Additional file 2: Additional Materials and Methods. gDNA isolation, Sanger sequencing confirmations of rips-1 alleles in DTT strains, total RNA extraction, 3′RACE for rips-1, and genotyping. Additional Results. 3′ untranslated region from rips-1 (R08E5.3). Table S1. DTT resistance alleles. Table S2. RNAi mini-screen for B12-related pathways. Table S3.C. elegans strains. Table S4. Oligonucleotide primers. Table S5. Plasmids. Additional References. [file 12915_2022_1415_MOESM2_ESM.docx]

**Additional File 2**

**Additional Materials and Methods**

**gDNA isolation**

Worms were grown on NGM OP50-1 plates and collected just prior to clearing of the bacterial lawn by washing with M9 buffer and collection in 15 mL tubes followed by centrifugation at 1150 xg for 3 minutes in an AccuSpin centrifuge at 20°C. Worms were washed twice by resuspension in 12 mL M9 followed by centrifugation, re-suspended in M9 and placed on a rocking platform in a 15 mL tube on its side at room temperature for 2 hours, washed twice with M9 and then the worm pellet frozen at -80°C. Genomic DNA was isolated using Gentra Puregene Tissue Kit (QIAGEN) by following the DNA Purification from Tissue protocol but without the liquid nitrogen grinding step. DNA was further purified using a Genomic DNA Clean & Concentrator kit (Zymo Research) and quantified using a Qubit dsDNA Broad Range Assay Kit (Thermo Fisher).

**Sanger sequencing confirmations of *rips-1* alleles in DTT strains**

gDNA was isolated, as described above, from the DTT resistant strains (non-Hawaiian crossed) and a 2429 bp of the *rips-1* locus amplified using *PfuUltra* II (Agilent) with primers oGS0001 and oGS0002**.** PCR products were cleaned using a QIAquick PCR Purification Kit (QIAGEN) and Sanger sequencing using primers oGS0001, oGS0002, oGS0003 and oGS0004. All Sanger sequencing in this study was performed by Eurofins Genomics.

**Total RNA extraction**

Worm pellets frozen at 80°C were re-suspended in 1 mL of TRIZOL Reagent (Invitrogen, ThermoFisher), added to a liquid nitrogen-cooled mortar and pestle then ground to a fine powder. Once thawed, samples were transferred to 1.5 mL tubes and frozen at 80°C. To isolate total RNA, samples were thawed, vortexed for 15 seconds, incubated for 10 minutes at room temperature with occasional vortexing, then centrifuged for 10 minutes at 12,000g at 4°C to pellet the insoluble material. Samples were transferred to 2 mL RNAse-free tubes (Ambion) and a Direct-zol RNA MiniPrep (Zymo Research) protocol with on-column DNase I treatment followed. Purified total RNA was quantified using a QUBIT RNA High Sensitivity Assay kit (Invitrogen, ThermoFisher) and RNA integrity assessed by agarose gel electrophoresis.

**3ʹRACE for *rips-1***

RNA was isolated from mixed stage and synchronised L4 cultures of wild type N2 as described above. A 3´RACE System for Rapid Amplification of cDNA Ends (Invitrogen, ThermoFisher: 18373019) was used following the manufactures protocol using 1 μg of total RNA from each preparation. PCR was performed on the 3ʹRACE material using GoTaq2 (Promega) with primers AUAP and oADW0254, with secondary amplification using a 1/1000 dilution of primary PCR as template with primers AUAP and oADW0255. PCR products were purified using QIAGEN PCR purification kit, cloned into pCR2.1TOPO and Sanger sequenced with M13Rev(-29), M13Uni(-21).

**Genotyping**

Single worms were lysed in 5 μL of a Proteinase K solution (0.5 μg/μL final concentration proteinase K; added immediately before use to a buffer containing 10 mM Tris (pH 8.0), 50 mM KCl, 2.5 mM MgCl2, 0.45% Tween-20 and 0.05% gelatine) by incubating for 1 hour at 65°C followed by 95°C for 15 minutes and the lysis reaction used directly as PCR template using GoTaq G2 Flexi DNA Polymerase (Promega). The DTT resistant amino acid substitution allele *rips-1 (ij109)* was genotyped by Sanger sequencing. Following genetic crosses and selection on 5 mM DTT, PCR using primers oGS0003 and oGS0002 on single-worm lysates was used to amplify *rips-1* genomic sequence flanking the *ij109* allele with the product purified using a QIAquick PCR Purification Kit (QIAGEN) and then Sanger sequenced using oGS0003 and oADW0279. The *mce-1* (*ok243*) deletion breakpoints were first defined by Sanger sequencing of a PCR product generated using primers mce-1ok243 seq IL and mce-1ok243 seq IR. *rips-1 (ij109)* [strain TP193] was crossed with *mce-1(ok243)* [strain RB512] to create the double mutant strain TP390. The *mce-1* deletion genotype was followed using primers *mce-1F*/*mce-1F2*/*mce-1R*, and with the *rips-1(ij109)* point mutation confirmed as described above. *rips-1 (ij109)* [TP193 strain] was crossed with *metr-1(ok521)* [strain RB755] to create the double mutant strain TP391 with the *metr-1(ok521)* allele being followed using primers oADW0326/ oADW0327/ oADW0328.

**Additional Results**

**3ʹ untranslated region from *rips-1 (R08E5.3)***

We noticed that the *rips-1* transcript is annotated in NCBI (NM_071644.5) and WormBase with 1112 bp 3ʹUTR. *cysl-3* (R08E5.2) is directly downstream of *rips-1* encoded on the opposite strand. The predicted 3ʹUTR of *rips-1* would therefore overlap significantly with coding regions of this neighbouring gene. To determine if this region of *rips-1* would be required for any expression construct and, due to the predicted functions of *cysl-3* in hydrogen sulphide interaction, if there might be a potential interaction between these two genes at the RNA level, 3ʹRACE was performed to experimentally define the 3ʹUTR for *rips-1*. Sequencing of 3ʹRACE products from mixed developmental stage and stage-specific (synchronised L4s) was analysed which revealed that the position of poly-A addition site for longest product identified is 177 nucleotides after the annotated stop. This results in an overlap region of only 76 bp involving only 12 bp if *cysl-3* coding sequence making any interaction at the RNA level unlikely.

**Additional Tables**

**Table S1. DTT resistance alleles**

| **Allele** | **Genomic co-ords (WS220)** | **Nucleotide change**  ***rips-1***  **NM_071644.5** | **Amino acid change**  **RIPS-1**  **NP_504045.1** | **Sequencing (strain used)** |
| --- | --- | --- | --- | --- |
| *ka13* | V: 3772286: G>A | c.49-1 G>A (caG/caA) | Exon 2 splice acceptor | WGS (TP173)  Sanger (TP278) |
| *ka14* | V: 3772286: G>A | c.49-1 G>A (caG/caA) | Exon 2 splice acceptor | WGS (TP174)  Sanger (TP251) |
| *gk902193(^a^)* | V: 3772550: G>A | c.264: tgG/tgA | p.W88* | Sanger (TP295) |
| *ij109* | V: 3773675: T>A | c.400 T>A (Tac/Aac) | p.Y134N | WGS (TP193)  Sanger (TP193) |
| *ka23* | V: 3773814: T>A | c.539 T>A (cTg/cAg) | p.L180Q | WGS (TP183)  Sanger (TP276) |
| *ka29* | V: 3773814: T>A | c.539 T>A (cTg/cAg) | p.L180Q | Sanger (TP189) |
| *ka18* | V: 3773823: G>A | c.548 G>A (gGg/gAg) | p.G183E | Sanger (TP178) |
| *ka28* | V: 3773874: C>T | c.599 C>T (tCg/tTg) | p.S200L | Sanger (TP188) |
| *ka22* | V: 3774050: C>T | c.775 C>T (Cag/Tag) | p.Q259* | Sanger (TP182) |
| *ka9* | V: 3774121: C>T | c.799 C>T (Caa/Taa) | p.Q267*  (p.P116L) | WGS (TP169)  Sanger (TP169) |
| *ka15* | V: 3774133: C>T | c.811 C>T (Cga/Tga) | p.R271*  (p.A150V) | WGS (TP175)  Sanger (TP252) |
| *ka20* | V: 3774245: C>T | c.923 C>T (tCc/tTc) | p.S308F | Sanger (TP180) |
| *ka11* | V: 3774287: C>T | c.965 C>T (gCt/gTt) | p.A322V | WGS (TP171)  Sanger (TP277) |
| *ka21* | V: 3774308: G>A | c.986 G>A (tGg/tAg) | p.W329* | WGS (TP181)  Sanger (TP279) |

^a^ *C. elegans* Million Mutation Project allele.

**Table S2. RNAi mini-screen for B12-related pathways**

| **Gene** | **Cosmid ID**  **(RNAi Library ID)** | **Description** | **Pathway** |
| --- | --- | --- | --- |
| *cblc-1* | ZK546.17  (II-10O13) | Ortholog of human MMACHC (methylmalonic aciduria (cobalamin deficiency) cblC type, with homocystinuria)  CblC. | B12 processing |
| _ | Y76A2B.5  (III-6B14) | Ortholog of MMADHC (methylmalonic aciduria and homocystinuria, cblD type)  CblD. | B12 processing |
| *sams-1* | C49F5.1  (X-5P21) | S-adenosylmethionine synthase | Methionine |
| *ahcy-1* | K02F2.2  (I-3G22) | S-AdenosylhomoCysteine HYdrolase homolog | Methionine |
| *metr-1* | R03D7.1  (II-7H13) | Methionine synthetase  CblG | Methionine |
| *mtrr-1* | C01G6.6  (II-6L07) | Methionine synthetase reductase  CblE | Methionine |
| *mthf-1* | C06A8.1  (II-5F12) | Orthologous to human methylenetetrahydrofolate reductase (MTHFR) | Folate |
| *mel-32* | C05D11.11  (III-3M14) | serine hydroxymethyltransferase | Folate |
| *cbs-1* | ZC373.1  (X-8C04) | CYSTATHIONINE-BETA-SYNTHASE | Transulpheration |
| *cysl-1* | C17G1.7  (X-4L22) | CYsteine Synthase Like | Transulpheration |
| *cysl-2* | K10H10.2  (II-9M07) | CYsteine Synthase Like | Transulpheration |
| *cysl-3* | R08E5.2  (V-3C18) | CYsteine Synthase Like | Transulpheration |
| *cbl-1* | C12C8.2  (I-4J09) | putative cystathionine gamma-lyase orthologous to human CTH | Transulpheration |
| *cth-1* | F22B8.6  (V-10P21) | putative cystathionine gamma-lyase; CTH-1 is orthologous to human CTH | Transulpheration |
| *cth-2* | ZK1127.10  (II-5I22) | putative cystathionine gamma-lyase; CTH-2 is orthologous to human CTH | Transulpheration |
| *gcs-1* | F37B12.2  (II-6D11) | C. elegans ortholog of gamma-glutamine cysteine synthetase heavy chain (GCS(h) | Transulpheration |
| *gss-1* | M176.2  (II-6P23) | C. elegans ortholog of glutathione synthetase (GSS) | Transulpheration |
| *mmaa-1* | T02G5.13  (II-5M04) | Methylmalonic aciduria type A protein | AdoCbl processing |
| *mmab-1* | C26E6.11  (III-2H11) | Methylmalonic aciduria type B protein, co(I)balamin adenosyltransferase (MMAB) | AdoCbl processing |
| *pcca-1* | F27D9.5  (X-8D01) | Propionyl-CoA carboxylase alpha chain | Canonical propanoic acid |
| *pccb-1* | F52E4.1  (X-2G13) | Propionyl-CoA carboxylase beta chain | Canonical propanoic acid |
| *mce-1* | D2030.5  (I-3B22) | Methylmalonyl-CoA epimerase | Canonical propanoic acid |
| *mmcm-1* | ZK1058.1  III-8D20 | Methylmalonyl-CoA mutase (MCM) | Mitochondrial B12 requiring enzyme |
| *acdh-1* | C55B7.4  (I-3I23) | Acyl CoA DeHydrogenase | Propanoic acid shunt |

**Table S3. *C. elegans* strains**

For the DTT resistance alleles ij109 and ka9, outcrossed strains were used both for mapping/WGS and for Sanger sequencing. For six DTT resistance alleles, non-outcrossed strains were used for mapping/WGS and outcrossed strains used for Sanger sequencing. For five alleles only Sanger sequencing was performed, done using non-outcrossed strains. For DTT resistance allele ka17 a possible large re-arrangement was indicated.

| **Strain** | **Genotype** | **Description** | **Source and references** |
| --- | --- | --- | --- |
| TP193 | *rips-1 (ij109)* V | DTT resistant mutant. 0utcrossed twice to N2.  Used for Hawaiian mapping/NGS.  Sanger sequenced for *rips-1*. | This study |
| TP169 | *rips-1 (ka9)* V | DTT resistant mutant. Outcrossed twice to N2.  Used for Hawaiian mapping/NGS.  Sanger sequenced for *rips-1*. | This study |
| TP171 | *rips-1 (ka11)* V | DTT resistant mutant. Not outcrossed.  Used for Hawaiian mapping/NGS. | This study |
| TP277 | *rips-1 (ka11)* V | DTT resistant mutant. Outcrossed twice to N2.  Sanger sequenced for *rips-1*. | This study |
| TP173 | *rips-1 (ka13)* V | DTT resistant mutant. Not outcrossed.  Used for Hawaiian mapping/NGS. | This study |
| TP278 | *rips-1 (ka13)* V | DTT resistant mutant. Outcrossed twice to N2.  Sanger sequenced for *rips-1*. | This study |
| TP174 | *rips-1 (ka14)* V | DTT resistant mutant. Not outcrossed.  Used for Hawaiian mapping/NGS. | This study |
| TP251 | *rips-1 (ka14)* V | DTT resistant mutant. Outcrossed twice to N2.  Sanger sequenced for *rips-1*. | This study |
| TP175 | *rips-1 (ka15)* V | DTT resistant mutant. Not outcrossed.  Used for Hawaiian mapping/NGS. | This study |
| TP252 | *rips-1 (ka15)* V | DTT resistant mutant. Outcrossed twice to N2.  Sanger sequenced for *rips-1*. | This study |
| TP181 | *rips-1 (ka21)* V | DTT resistant mutant. Not outcrossed.  Used for Hawaiian mapping/NGS. | This study |
| TP279 | *rips-1 (ka21)* V | DTT resistant mutant. Outcrossed twice to N2.  Sanger sequenced for *rips-1*. | This study |
| TP183 | *rips-1 (ka23)* V | DTT resistant mutant. Not outcrossed.  Used for Hawaiian mapping/NGS. | This study |
| TP276 | *rips-1 (ka23)* V | DTT resistant mutant. Outcrossed twice to N2.  Sanger sequenced for *rips-1*. | This study |
| TP178 | *rips-1 (ka18)* V | RIPS-1 DTT resistant mutant, not outcrossed.  Sanger sequenced for *rips-1*. | This study |
| TP180 | *rips-1 (ka20)* V | DTT resistant mutant, not outcrossed.  Sanger sequenced for *rips-1*. | This study |
| TP182 | *rips-1 (ka22)* V | DTT resistant mutant. Not outcrossed.  Sanger sequenced for *rips-1*. | This study |
| TP188 | *rips-1 (ka28)* V | DTT resistant mutant. Not outcrossed.  Sanger sequenced for *rips-1*. | This study |
| TP189 | *rips-1 (ka29)* V | DTT resistant mutant. Not outcrossed.  Sanger sequenced for *rips-1.* | This study |
| TP177 | *ka17* | DTT resistant mutant. Not able to outcross.  Used for Hawaiian mapping/NGS. Mapping to chromosome I, possible large re-arrangement. No variant in *rips-1* found by NGS or Sanger sequencing. |  |
| VC40962 | *rips-1* (gk902193) V | *C. elegans* Million Mutation Project strain | Caenorhabditis Genetics Center (CGC) |
| TP295 | *rips-1* (gk902193) V | Outcrossed 4 times to N2, selected on DTT.  Sanger sequenced for *rips-1*. | This study |
| TP313 | *rips-1 (ij109)* V; kaEx [pLBG007 (RIPS-1::GFP) + pADW021(3) (P*myo-2*::mCherry)] | DTT resistant strain carrying RIPS-1::GFP extrachromosomal array | This study |
| TP315 | *rips-1(ij109)* V; kaEx [pLBG007 (RIPS-1::GFP) + pADW021(3) (*Pmyo-2*::mCherry)] | DTT resistant strain carrying RIPS-1::GFP extrachromosomal array | This study |
| VL749 | wwIs24 [*acdh-1p*::GFP + unc-119(+)] | Propionic acid marker | CGC. [1] |
| RB755 | R03D7.1(ok521) II | Methionine synthetase (*metr-1*) mutant | CGC. [2] |
| RB512 | D2030.5(*ok243*) I | Methylmalonyl-CoA Epimerase (*mce-1*) mutant | CGC. [2] |
| TP390 | D2030.5(ok243) I; *rips-1* (ij109) V. | *rips-1* and Methylmalonyl-CoA Epimerase (*mce-1*) double mutant | This study |
| TP391 | *metr-1(ok521)* II; *rips-1* (ij109) V] | *rips-1* and Methionine synthetase (*metr-1*) double mutant | This study |

**Table S4. Oligonucleotide primers**

Sequences are shown 5′-3′. Engineered bases are underlined and restriction enzymes sites are shown in bold font. All oligonucleotide primers were synthesised at Eurofins Genomics. Primers for qPCR and site-directed mutagenesis were HPLC purified.

| **Genotyping primers for a 2940 bp deletion on chromosome V of the Hawaiian strain CB4856** | | |
| --- | --- | --- |
| oADW0165 | GGGATCACCATATTTGGTAAGA (22) | F1 primer, flanks deletion. |
| oADW0166 | CATCGTGATGAAAAGTTGATGAC (23) | R1 primer, flanks deletion. |
| oADW0168 | CCAGTAATGCTTCAGACAAGT (21) | F2 primer, internal to deletion. |

| **Amplification and Sanger sequencing of *rips-1* genomic locus** | | |
| --- | --- | --- |
| oGS0001 | CTACACAACACGTGGACAAC (20) | Amplification forward and sequencing primer. |
| oGS0002 | GTATTCCCCAGCCAGCCATG (20) | Amplification reverse and sequencing primer. |
| oGS0003 | GTAATCGTGAGGTACTCATAC (21) | Sequencing primer. |
| oGS0004 | GTAATGGAACAATCTGACAC (20) | Sequencing primer. |

| **Amplification and Sanger sequencing of allele *rips-1* (*ij109)* for confirmation of genotype after crossing** | | |
| --- | --- | --- |
| oGS0003 | GTAATCGTGAGGTACTCATAC (21) | Amplification forward primer. |
| oGS0002 | GTATTCCCCAGCCAGCCATG (20) | Amplification reverse primer. |
| oADW0279 | GGAGTCCGTCCAGATTTCTG (20) | Sequencing primer. |

| ***rips-1* 3’RACE** | | |
| --- | --- | --- |
| oADW0254 | GGATGTTCGCTATGGTAGAG (20) | Forward primer for 3’RACE. |
| oADW0255 | GGCTCCAGTAACGTATACAC (20) | Nested forward primer for 3’RACE. |
| AUAP | GGCCACGCGTCGACTAGTAC (20) | Abridged universal amplification primer. |

| **RIPS-1::GFP translational fusion** | | |
| --- | --- | --- |
| oADW0256 | GCAGGTAGGCAGGCATAGAA (20) | *rips-1* amplification forward primer. includes a small amount of R08E5.1 coding sequence. |
| oADW0258 | CGATGAGGCGATGGGAATTG (20) | *rips-1* amplification reverse primer. Designed downstream of longest experimental (3’RACE from this study) 3’UTR for *rips-1* and just into 3’ end of *cysl-3* coding sequence |
| oADW0272 | GTATTGTGCCCAGAAAAA**CCC**GGGTACTATTGAAGCC (37) | Site-directed mutagenesis forward primer. Used on *rips-1* rescue construct to mutate stop codon and introduce and an *Xma* I restriction site. |
| oADW0273 | GGCTTCAATAGTACCC**GGG**TTTTTCTGGGCACAATAC (37) | Site-directed mutagenesis reverse primer. |
| oADW0274 | AA**CCCGGG**TATGAGTAAAGGAGAAGAACTTTTCAC (35) | GFP amplification forward primer with *Xma* I restriction site. Additional “T” to adjust reading frame for insertion after R08E5.3. |
| oADW0275 | AA**CCCGGG**CTATTTGTATAGTTCATCCATGCCAT (34) | GFP amplification reverse primer with *Xma* I restriction site |
| oADW0254 | GGATGTTCGCTATGGTAGAG (20) | Designed for 3’RACE, also used to sequence site-directed mutagenized plasmids over the engineered change. |

| **qRT-PCR (one primer of pair was designed over an exon-exon boundary to reduce signal from residual gDNA).** | | |
| --- | --- | --- |
| oADW0306 | AGGCTGCAAAGAGAGGTACA (20) | R08E5.3 F. Designed to be specific for *rips-1* and should not amplify paralog R08E5.1, K12D9.1 or R08F11.4 |
| oADW0307 | TCGTCGAGAGGCTCCAAC (18) | R08E5.3 R. Designed to be specific for *rips-1* and should not amplify paralogs |
| oADW0308 | CACGTGGAGAATGTGAAGGAC (21) | R08E5.1 F. Designed to be specific for R08E5.1 |
| oADW0309 | GCTTCTCGACAACTCCGTGA (20) | R08E5.1 R. Designed to be specific for R08E5.1 |
| oADW0114 | GCCAACGATCAAGGAAACAG (20) | hsp-4 (F43E2.8) F |
| oADW0115 | GATCCAACCTTCACCTCAAC (20) | hsp-4 (F43E2.8) R |
| oADW0312 | GAGGTTCAAAAGGACTTAAAGG (22) | hsp-6 (C37H5.8) F |
| oADW0313 | GTAGCTTGACGCTGAGAATC (20) | hsp-6 (C37H5.8) R |
| oADW0314 | CACTATGGGCCCAAAAGGAA (20) | hsp-60 (Y22D7AL.5) F |
| oADW0315 | CTTGACGAATGCTCTCGAATC (21) | hsp-60 (Y22D7AL.5) R |
| oADW0322 | GCCCACACAAAATTCAAGGCAT (22) | cysl-2 (K10H10.2) F |
| oADW0323 | GTCGCTTGGCCAACTGAAC (19) | cysl-2 (K10H10.2) R |
| oADW0320 | GGCTATCGCTTGCAAAGCAT (20) | nhr-57 (T05B4.2) F |
| oADW0321 | GGCTTGTTGGATTGCTTGAAC (21) | nhr-57 (T05B4.2) R |
| oADW0118 | AAGATCTATTGTTCTACCAGGC (22) | tbg-1 (F58A4.8, tubulin gamma). Primer from [3] |
| oADW0119 | CTTGAACTTCTTGTCCTTGAC (21) | tbg-1 (F58A4.8, tubulin gamma) R. Primer from [3] |
| oADW0310 | GCTCTTGCCCCATCAACC (18) | act-3 (T04C12.4, actin) F |
| oADW0311 | GAGAAAATGGTAAGGCGAAGAG (22) | act-3 (T04C12.4, actin) R |

| **Genotyping *mce-1* and *metr-1* mutant strains** | | |
| --- | --- | --- |
| mce-1ok243 seq IL | CCAAGTAGCCTTCATCTCGC (20) | *mce-1* (D2030.5) allele *ok243*. Primer to define deletion. Primer from [https://cgc.umn.edu/strain/RB512](about:blank) |
| mce-1ok243 seq IR | CCCATGTGCGTAAGGAATTT (20) | *mce-1* (D2030.5) allele *ok243*. Primer to define deletion. Primer from [https://cgc.umn.edu/strain/RB512](about:blank) |
| *mce-1F* | GCAAGTTTACAGCGGGTTCATTG (23) | mce-1 genotyping primer F1 |
| mce-1F2 | AAGCGAAGAATGTCAATAATCAG (23) | mce-1 genotyping primer F2 |
| mce-1R | GAGACTGAACGATGTTGAATAAC (23) | mce-1 genotyping primer R1 |
| oADW0326 | CGAGGATGAAGGAGTTCCAG (20) | *metr-1* (R03D7.1) allele ok521 genotyping F1. Primer from [https://cgc.umn.edu/strain/RB755](about:blank) |
| oADW0327 | GATGAAAGAATCAGACCACTTCG (23) | *metr-1* (R03D7.1) allele ok521 genotyping R1 |
| oADW0328 | TAAACTTGTCCCAGTCGATCTTG (23) | *metr-1* (R03D7.1) allele ok521 genotyping R2 |

**Table S5. Plasmids**

| **Name** | **Description** |
| --- | --- |
| pADW0102(1) | *rips-1* 3’RACE mixed stage, pCR2.1 TOPO |
| pADW0103(12) and (17) | *rips-1* 3’RACE L4, pCR2.1 TOPO |
| pADW0105 | *rips-1* (+), pCR2.1TOPO |
| pLBG003 | *rips-1* (*Xma* I), pCR2.1TOPO |
| pLBG004 | GFP (*Xma* I/*Xma* I), pCR-Blunt II-TOPO |
| pLBG007 | *rips-1* *^prom^*::RIPS-1::GFP::RIPS-1 |

**Additional references**

1. MacNeil LT, Watson E, Arda HE, Zhu LJ, Walhout AJ. Diet-induced developmental acceleration independent of TOR and insulin in C. elegans. Cell 2013, 153(1):240-252.

2. Consortium CeDM. large-scale screening for targeted knockouts in the Caenorhabditis elegans genome. G3 (Bethesda) 2012, 2(11):1415-1425.

3. Greiss S, Schumacher B, Grandien K, Rothblatt J, Gartner A. Transcriptional profiling in C. elegans suggests DNA damage dependent apoptosis as an ancient function of the p53 family. BMC Genomics 2008, 9:334.
